# Md-miR156ab and Md-miR395 Target WRKY Transcription Factors to Influence Apple Resistance to Leaf Spot Disease

**DOI:** 10.3389/fpls.2017.00526

**Published:** 2017-04-19

**Authors:** Qiulei Zhang, Yang Li, Yi Zhang, Chuanbao Wu, Shengnan Wang, Li Hao, Shengyuan Wang, Tianzhong Li

**Affiliations:** Laboratory of Fruit Cell and Molecular Breeding, China Agricultural UniversityBeijing, China

**Keywords:** apple, apple leaf spot, miRNAs, WRKY TF

## Abstract

MicroRNAs (miRNAs) are key regulators of gene expression that post-transcriptionally regulate transcription factors involved in plant physiological activities. Little is known about the effects of miRNAs in disease resistance in apple (*Malus*×*domestica*). We globally profiled miRNAs in the apple cultivar Golden Delicious (GD) infected or not with the apple leaf spot fungus *Alternaria alternaria f. sp. mali* (ALT1), and identified 58 miRNAs that exhibited more than a 2-fold upregulation upon ALT1 infection. We identified a pair of miRNAs that target protein-coding genes involved in the defense response against fungal pathogens; Md-miR156ab targets a novel WRKY transcription factor, MdWRKYN1, which harbors a TIR and a WRKY domain. Md-miR395 targets another transcription factor, MdWRKY26, which contains two WRKY domains. Real-time PCR analysis showed that Md-miR156ab and Md-miR395 levels increased, while *MdWRKYN1* and *MdWRKY26* expression decreased in ALT1-inoculated GD leaves; furthermore, the overexpression of Md-miR156ab and Md-miR395 resulted in a significant reduction in *MdWRKYN1* and *MdWRKY26* expression. To investigate whether these miRNAs and their targets play a crucial role in plant defense, we overexpressed *MdWRKYN1* or knocked down Md-miR156ab activity, which in both cases enhanced the disease resistance of the plants by upregulating the expression of the WRKY-regulated pathogenesis-related (PR) protein-encoding genes *MdPR3-1, MdPR3-2, MdPR4, MdPR5, MdPR10-1*, and *MdPR10-2*. In a similar analysis, we overexpressed *MdWRKY26* or suppressed Md-miR395 activity, and found that many PR protein-encoding genes were also regulated by *MdWRKY26*. In GD, ALT-induced Md-miR156ab and Md-miR395 suppress *MdWRKYN1* and *MdWRKY26* expression, thereby decreasing the expression of some PR genes, and resulting in susceptibility to ALT1.

## Introduction

MicroRNAs (miRNAs) are non-coding RNA molecules of around 20–24 nt in length, which bind to complementary sequences in messenger RNAs (mRNAs) to induce their degradation or to inhibit their translation, resulting in the silencing of the corresponding gene (Llave et al., 2002; Bartel, 2004; Baulcombe, 2004). MiRNAs are crucial regulatory factors of almost all biological processes, including plant growth, development, and abiotic stress responses (Sunkar et al., 2007; Padmanabhan et al., 2009; Rubio-Somoza et al., 2009; Zhang et al., 2011). The first group of miRNAs to be identified in plants included miR171, which accumulates predominantly in the inflorescence tissues of *Arabidopsis thaliana* and targets several members of the SCARECROW-LIKE (SCL) family of transcription factors, which is involved in gibberellin-regulated chlorophyll biosynthesis (Llave et al., 2002). Since then, many researchers have identified miRNAs that silence transcription factors to regulate diverse biological processes in plants; for example, miRNA156 targets members of the SQUAMOSA-PROMOTER BINDING PROTEIN-LIKE (SPL) transcription factors that regulate the transition from vegetative to reproductive growth, tillering/branching, panicle/tassel architecture, and response to abiotic stresses in *Arabidopsis* (Wu and Poethig, 2006; Wang et al., 2009; Gou et al., 2011; Stief et al., 2014; Wang and Wang, 2015). Another miRNA, miRNA159, represses MYB transcription factors, which participate in gibberellin (GA)-induced pathways required for aleurone development and cell death (Reyes and Chua, 2007; Alonso-Peral et al., 2010). MiRNA159 also degrades *MYB33* and *MYB101* transcripts to desensitize *Arabidopsis* seedlings to hormone signaling during stress responses (Reyes and Chua, 2007; Alonso-Peral et al., 2010). Thus, miRNAs regulate diverse families of transcription factors and play essential roles in the signaling cascades of various biological processes; however, few studies have investigated the ability of miRNAs to regulate transcription factors in biotic stress pathways in plants.

In response to pathogen invasion, the pathogen-associated molecular pattern (PAMP)- and effector-triggered immunity (PTI and ETI) pathways are induced in the host plant. Pathogens can respond with a diverse array of virulence factors that suppress host defenses. Plant resistance genes such as those encoding the intracellular resistance (R) protein receptors can recognize these virulence effectors, often triggering a hypersensitive cell death response (Gus-Mayer et al., 1998; Ausubel, 2005; Jones and Dangl, 2006; Dodds and Rathjen, 2010; Vargas et al., 2012; Meng et al., 2013; Teixeira et al., 2014). Previous studies have shown that several members of the WRKY transcription factor (TF) family, named from a highly conserved WRKY domain, are targeted by R proteins, which activate them to recognize W-box elements in the promoters of pathogenesis-related (PR) proteins (Rushton et al., 1996; Eulgem et al., 1999, 2000). WRKY TFs include the WRKYGQK motif followed by a Cx4–5Cx22–23HxH or Cx7Cx23HxC zinc-finger motif (Rushton et al., 2010; Chi et al., 2013; Schluttenhofer and Yuan, 2015; Phukan et al., 2016). In *Arabidopsis* and *Oryza sativa* (rice), most WRKY genes are implicated in defense (Dong et al., 2003; Hwang et al., 2011; Matsushita et al., 2013).

Golden Delicious (GD) is a commonly cultivated cultivar of apple (*Malus*×*domestica*). It is highly susceptible to the pathogenic fungus *Alternaria alternaria f. sp. mali* (ALT1), which causes leaf blotch and fruit spot. The current management of *Alternaria* blotch primarily uses traditional chemical control agents instead of resistant cultivars; however, a new approach to solve this problem would be to target the molecular mechanisms involved in the process of pathogen resistance in apple (Zhang et al., 2015). While the function of WRKY proteins in plant defense and PR regulation is well-documented in *Arabidopsis* and rice, these transcription factors have not previously been investigated in apple. A recent study identified 127 potential WRKY-encoding genes, some of which were believed to be involved in the biotic and abiotic stress responses (Meng et al., 2016). Several conserved miRNAs in apple have been predicted to target transcription factors based on expressed sequence tag (EST) sequences (Xia et al., 2012). A recent study reported a direct correlation between the accumulation of miR396 and the silencing of WRKY expression in sunflower *(Helianthus annuus;* Giacomelli et al., 2012), which suggested a similar relationship might be found in other species, including apple. In the present study, a miRNA high-seq assay was performed in GD, leading to the identification of two miRNAs, Md-miRNA156ab and Md-miRNA395, which target two different WRKY transcription factors to regulate the resistance of apple to the pathogenic ALT1 fungus.

## Materials and methods

### Plant materials and growth conditions

Apple (*Malus*×*domestica* cv. “Golden Delicious,” GD) plants were grown in tissue culture on Murashige and Skoog medium containing 0.6 mg·L^−1^ 6-benzylaminopurine and 0.15 mg·L^−1^ 1-naphthylacetic acid in a climate-controlled culture room at 24 ± 1°C, with a 16/8 h photoperiod. Four-week-old plants were used for *Agrobacterium tumefaciens* infiltration and fungal infection experiments.

### *Agrobacterium tumefaciens* infiltration

Full-length *Md-MIR156ab, Md-MIR395, STTM-miR156ab, STTM-miR395, MdWRKYN1*, and *MdWRKY26* gene sequences were individually inserted into the plant expression vector pFGC5941 (GenBank AY310901) at the NcoI/BamHI restriction site (primers listed in Supplemental Table 3), using the empty pFGC5941 vector as the control. The vectors were transformed into *Agrobacterium tumefaciens* (strain GV3101) using the heat shock transformation method. Plants of the susceptible apple variety GD were infiltrated by *Agrobacterium tumefaciens* containing one miRNA gene, performed when the fifth leaf of the seedling had expanded at approximately 4-weeks-old, as described previously (Bai et al., 2011).

### Fungal growth and infection assay

A plant-pathogenic ALT1 (*Alternaria alternata f. sp. mali*) strain was grown on a potato dextrose agar medium at 25°C for 6 days. Inoculum spores were suspended in deionized water at 2 × 10^5^ CFU/ml, quantified using microscopy. Apple leaves from 4-week-old plants were inoculated by swabbing them with a spore suspension of the ALT1 pathogen (Bai et al., 2011), 4 days after *Agrobacterium tumefaciens* infiltration (Ma et al., 2014).

### Small-RNA library construction and next-generation sequencing

Small RNA was isolated from 4-week-old plants of the ALT1-susceptible GD apple cultivar at 24 hpi with ALT1, or after a mock (control) inoculation. The small RNA libraries for Next-Generation sequencing were constructed by the 5′-phosphate-dependent method as described (Chellappan and Jin, 2009), and the libraries were sequenced using the Illumina HiSeq 2000 platform.

### Bioinformatic analysis of cloned sRNAs and known miRNA of apple

The 20–24-nt small RNA clones were compared with the miRBase database (www.mirbase.org/) to identify novel miRNA sequences. Then, miRNAs in miRBase and our potentially novel miRNA were compared with the apple genome (https://www.rosaceae.org/species/malus/all) by SOAP (Simple Object Access Protocol, http://soap.genomics.org.cn/soap1/). RNA secondary structure of the candidate sequences (http://mfold.rna.albany.edu/) was analyzed according to the method of Xie et al. (2010), followed by screening against the apple protein library (http://genomics.research.iasma.it/, 3,817 sequences) and CDS sequence library (http://genomics.research.iasma.it/, 336,889 sequences), to remove coding sequence.

### Identification and cloning of Md-miRNA-targeted genes

Putative target genes of the miRNAs were identified by aligning the sequence of the miRNAs to the sequence of the apple genome, using the Plant Small RNA Target Analysis Server (http://plantgrn.noble.org/psRNATarget/). Detailed annotation information about the targeted genes, including their nucleotide sequences, chromosome locations, and predicted protein domains, was obtained from the Genome Database for Rosaceae (https://www.rosaceae.org) and the National Center for Biotechnology Information genomic database (http://www.ncbi.nlm.nih.gov). Specific primers for *MdWRKYN1* and *MdWRKY26* are listed in Supplemental Table 4.

### Real-time PCR assay of mature miRNA

Leaf samples were harvested from control and ALT1-infected plants at 24 hpi, frozen in liquid nitrogen, and stored at −80°C until required. RNA was extracted from 100 mg of leaf tissue using the cetyltrimethylammonium bromide (CTAB) method (Gambino et al., 2008). The concentration of RNA was determined using an ND-1000 NanoDrop spectrophotometer (Thermo Fisher Scientific, USA). DNase-treated RNA (1 μg) was used in the reverse-transcription reaction and subsequent PCR amplification. A 1:1 ratio of miRNA and reference gene reverse-transcription primers (Supplemental Table 3) were used for the reverse-transcription reaction (Feng et al., 2009). Real-time PCR was performed using SuperReal PreMix Plus (Tiangen Biotech Co., Ltd., China), with 40 cycles of 95°C for 10 s and 60°C for 30 s performed using an Applied Biosystems 7500 (Thermo Fisher Scientific, USA). The relative abundance of the miRNAs was calculated using the 2^−ΔΔCT^ method (Livak and Schmittgen, 2001), normalized using *5S rRNA* as the reference gene (Ma et al., 2014). Specific primers for the mature miRNAs and *5S rRNA* are listed in Supplemental Table 3.

### Real-time PCR of Md-miRNA-target genes and PR genes

Total RNA was extracted from apple leaves using an EASY Spin Kit (Beijing Biomed Biotechnology Co., Ltd., China), amplified using oligo-dT primers (Takara Biomedical Technology Co., Ltd., China), and reverse-transcribed into cDNA (see Supplemental Table 3 for primers). Real-time PCR was performed using SuperReal PreMix Plus (SYBR Green) and the conditions outlined above. The relative RNA abundance was calculated using the 2^−ΔΔCT^ method (Livak and Schmittgen, 2001), using *MdActin* (NCBI XM_008365636.2) as the reference gene. Specific primers for *MdWRKYN1, MdWRKY26*, the 12 *PR* genes, and *MdActin* are listed in Supplemental Table 4.

### Statistical analysis of real-time PCR

For the real-time PCR, a total of nine leaves from three different plants subjected to the same treatment were collected as one sample for RNA isolation, reverse transcription, and real-time PCR. The same numbers of non-infiltrated GD seedlings and EV (pFGC5941)-infiltrated GD plants served as controls. The three real-time PCR reactions were regarded as three technical repeats for each sample. The same experiment was performed a total of three times for the *t*-test analysis.

### Statistical analysis of the disease rate

To calculate the disease rate, about 30 leaves from 10 GD apple seedlings were infiltrated by *Agrobacterium tumefaciens*. The same numbers of non-infiltrated GD seedlings and EV (pFGC5941)-infiltrated GD plants served as controls. All plants were inoculated with a spore suspension of the ALT1 pathogen. The disease rate in the inoculated seedlings was calculated at 24 hpi. These experiments were performed a total of three times, and a *t*-test was performed for the statistical analysis.

## Results

### Identification of ALT1-induced miRNAs by next-generation sequencing

**Figure 1:**
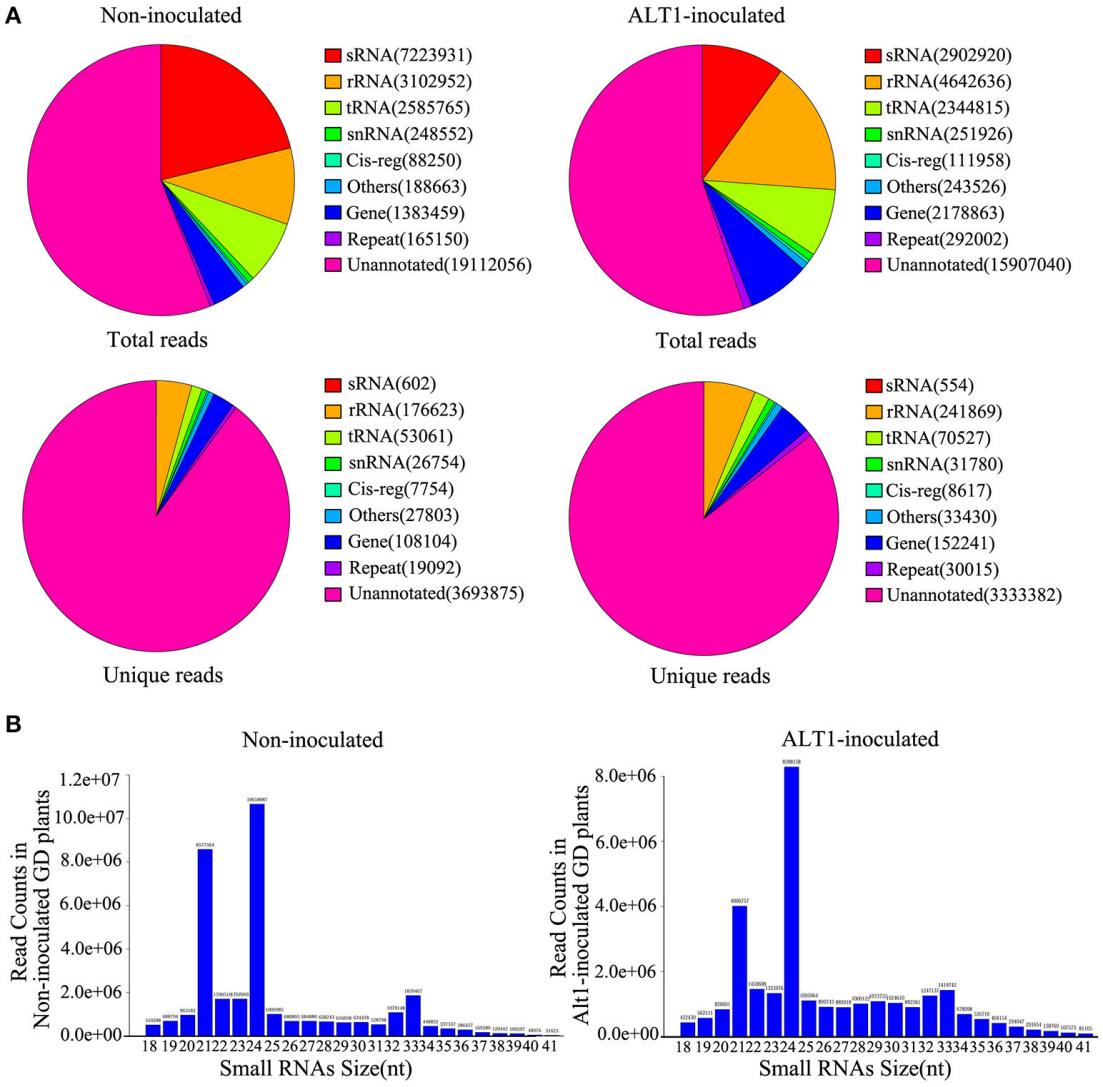
**Distribution of lengths and classification of small RNAs (sRNAs) in Golden Delicious (GD) apple leaves. (A)** Classification of sRNAs identified in plants inoculated with ALT1, relative to the control. rRNA, ribosomal RNA; repeat, repeat-associated RNA; snRNA, small nuclear RNA; Cig-reg, cis-regulatory modules; tRNA, transfer RNA; sRNA, small RNA. **(B)** Length distribution of sequence reads and unique data that perfectly map to previously identified miRNAs.

To examine apple miRNA accumulation in response to infection by ALT1, we performed next-generation sequencing of small RNAs (sRNAs). RNA was isolated from 4-week-old plants of the ALT1-susceptible GD cultivar of apple at 24 h post-inoculation (hpi) with ALT1, or after a mock (control) inoculation. After removing the poor-quality reads and adapter sequences, we obtained 34,098,778 high-quality clean reads from the control GD leaves and 28,875,686 clean reads from ALT1-inoculated plants. Unique reads were aligned to the genome of Malus × domestica. For annotation purposes, the sRNAs were classified into several classes (Figure 1A). The 24- and 21-nt sRNA species were the first and second most frequently observed lengths, respectively, in both the ALT1- and non-inoculated leaves (Figure 1B).

We identified miRNAs by performing a homology analysis on the sRNA sequences, using the following selection criteria; a length of at least 18 nt, and a maximum of two mismatches compared with the miRNA databases (miRBase and plant microRNA database) for the apple genome. The selected sequences were screened for predicted secondary structures matching miRNA standards, which resulted in a list of 319 and 308 previously identified miRNAs in the non-inoculated and ALT1-inoculated apple leaves, respectively, also according to the current understanding of miRNA composition and on base distribution data (Supplemental Figure 1). The majority of the sRNAs were unannotated. We mapped the unknown sRNAs to the reference sequence to identify novel miRNAs using Mireap software. The stem-loop structures matched to putative miRNA precursors were used to predict the characteristic fold-back RNA secondary structure, resulting in the identification of 56 potentially novel apple miRNAs. Although some of the novel miRNAs identified exists in other species, they had not hitherto been reported in apple. Therefore, we consider these to be novel miRNAs in apple.

**Figure 2:**
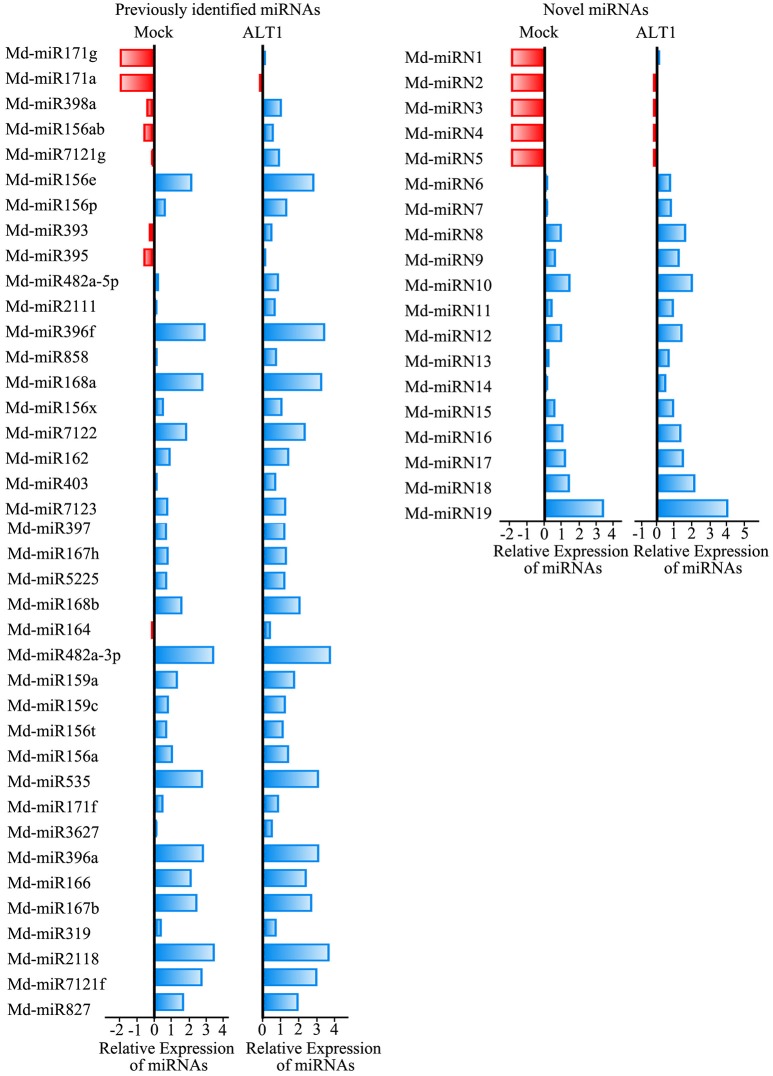
**Differentially expressed miRNAs identified in ALT1-inoculated and non-inoculated apple leaves**. Bars are log (base 2) ratios of fold changes relative to the mock. miRNAs were selected based on exhibiting at least a 2-fold change in abundance at 24 hpi. The blue bars represent increases in the expression levels of the miRNAs, while the red bars represent lower miRNA expression levels.

Among the sequenced miRNAs, we identified 39 previously identified miRNAs/miRNA families (Supplemental Table 1) and 19 novel miRNAs (Supplemental Table 2) that exhibited at least a 2-fold difference in expression level following ALT1 infection (Figure 2). Plant miRNAs bind almost perfectly to the complementary sequences of their target genes, regulating their post-transcriptional processing by inducing transcript degradation or translational inhibition. Identifying and validating the targets of the miRNAs is therefore important for elucidating their potential biological function. A total of 578 and 209 candidate target transcripts were identified for the previously identified miRNAs/miRNA families and novel miRNAs, respectively.

### Prediction and analysis of the targets of differentially expressed miRNAs

We conducted Gene Ontology (GO) and Kyoto Encyclopedia of Genes and Genomes (KEGG) pathway analyses to better understand these differentially expressed miRNAs and their target genes. According to the GO classification, the category containing the most target genes was “biological process,” which is sub-categorized into the 20 biological processes displayed in Supplemental Figure 2. In particular, the defense response (GO: 0006952), signal transduction (GO: 0007165), transcription factor (GO: 0006355), and oxidation-reduction processes (GO: 0055114) were significantly enriched (Supplemental Figure 2A). The KEGG-based analysis linked 39 miRNAs/miRNA families and 578 target genes to 42 pathways, with a significant enrichment of the plant hormone signal transduction (mdm04075), plant–pathogen interaction (mdm04626), and mRNA surveillance (mdm03015) pathways (Supplemental Figure 2B). These two analyses indicated that transcription factors play a crucial role in the response to ALT1 infection in GD leaves.

**Figure 3:**
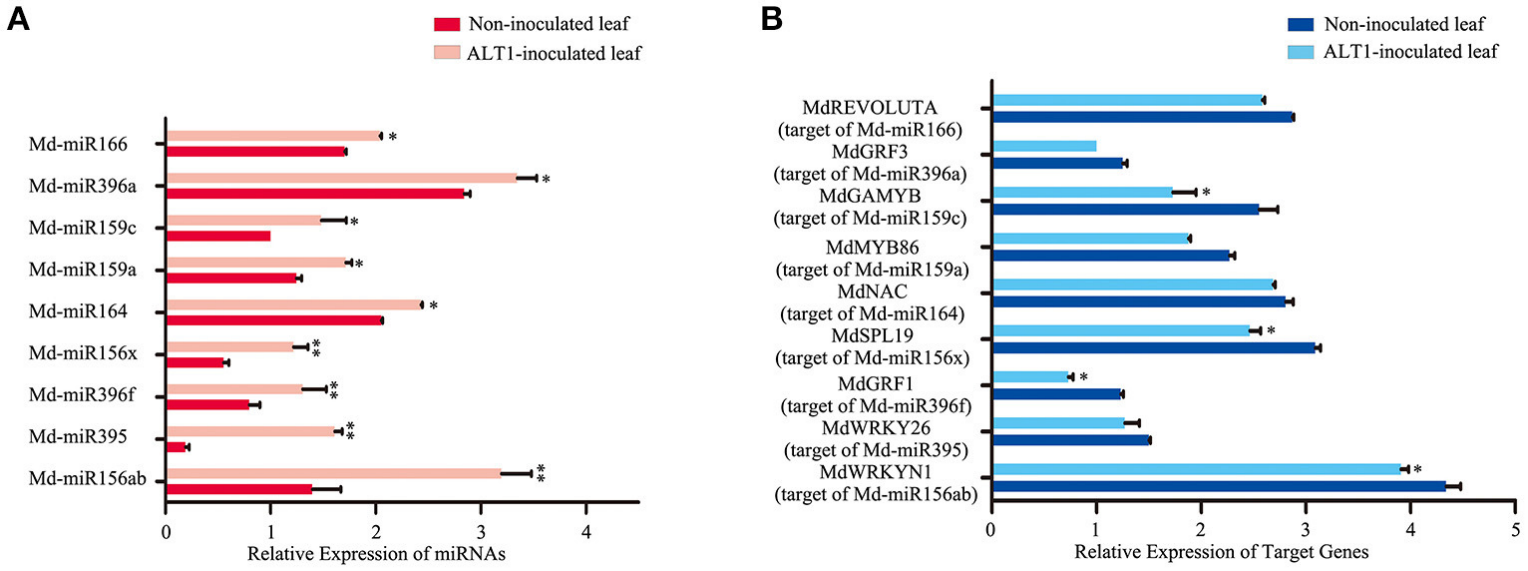
**Real-time PCR quantification of the differentially expressed microRNAs and their putative target transcription factors from infected and control plants at 24 hpi. (A)** Expression of differentially expressed miRNAs in ALT1-inoculated and control Golden Delicious (GD) apple leaves. **(B)** Expression of corresponding miRNA target genes in ALT1-inoculated and control GD leaves. Spore inoculum concentration: 2 × 10^5^ CFU/ml. Spore growth was measured at 6 dpi. Real-time PCR data were calculated based on three biological and three technical replicates. Error bars = SD; ^**^P < 0.01; ^*^P < 0.05 (Student's *t*-test).

Notably, several miRNAs that putatively regulate transcription factors were differentially expressed under ALT1 infection, and were therefore investigated in greater depth (Supplemental Figure 3). Md-miR156 family targets several genes encoding members of the SPL transcription factor family, which are reported to participate in the response to abiotic stress (Stief et al., 2014; Wang and Wang, 2015). In our libraries of differentially expressed miRNAs, one member of the Md-miR156 family, Md-miR156x (miRBase MIMAT0025890; miRBase website: http://www.mirbase.org/), showed a 3.78-fold change in abundance following ALT1 infection (Figures 2, 3). This suggests a potential role for the Md-miR156x target gene *MdSPL19* (NCBI XM_008343068.1; NCBI website: https://www.ncbi.nlm.nih.gov/) in plant immunity. Another Md-miR156 paralog, like Md-miR156a (miRBase MIMAT0025867), had a smaller change in relative expression than Md-miR156x, which also targeted a member of the SPL family (Figure 2). The expression of Md-miR164 (miRBase MIMAT0025908), which targets a NAC-domain transcription factor, *MdNAC* (NCBI XM_008376341.1), displayed a 4.35-fold upregulation at 24 hpi with ALT1, which functions as a stress-responsive transcription factor (Puranik et al., 2012; Zhu et al., 2014; Figures 2, 3). Md-miR166 (miRBase MIMAT0025913), which showed a 2.32-fold higher accumulation in the ALT1-infected apple leaves than in the leaves of the control plants, targets the homeobox-leucine zipper protein *MdREVOLUTA* (NCBI XM_008346650.2; Figures 2, 3). Md-miR159a (miRBase MIMAT0025898; 2.99-fold upregulation after ALT1 infection) and Md-miR159c (miRBase MIMAT0026053*;* 2.88-fold upregulation after ALT1 infection) target the MYB transcription factor genes *MdMYB86* (NCBI XM_008387744.1) and *MdGAMYB* (NCBI XM_008348120.1), respectively. The MYB TFs reported in *Arabidopsis* are also regulated by miR159 and function in plant growth and the response to stress (Reyes and Chua, 2007; Alonso-Peral et al., 2010; Figures 2, 3). Md-miR396a (miRBase MIMAT0025989) and Md-miR396f (miRBase MIMAT0025994), encoded by genes with two nucleotide differences, regulate conserved targets *MdGRF1* (NCBI XM_008343874.2) and *MdGRF3* (NCBI XM_008345268.1), which belong to the *GROWTH-REGULATING FACTOR (GRF)* family of transcription factors expressed during growth and stress responses (Debernardi et al., 2012; Figures 2, 3).

**Figure 4:**
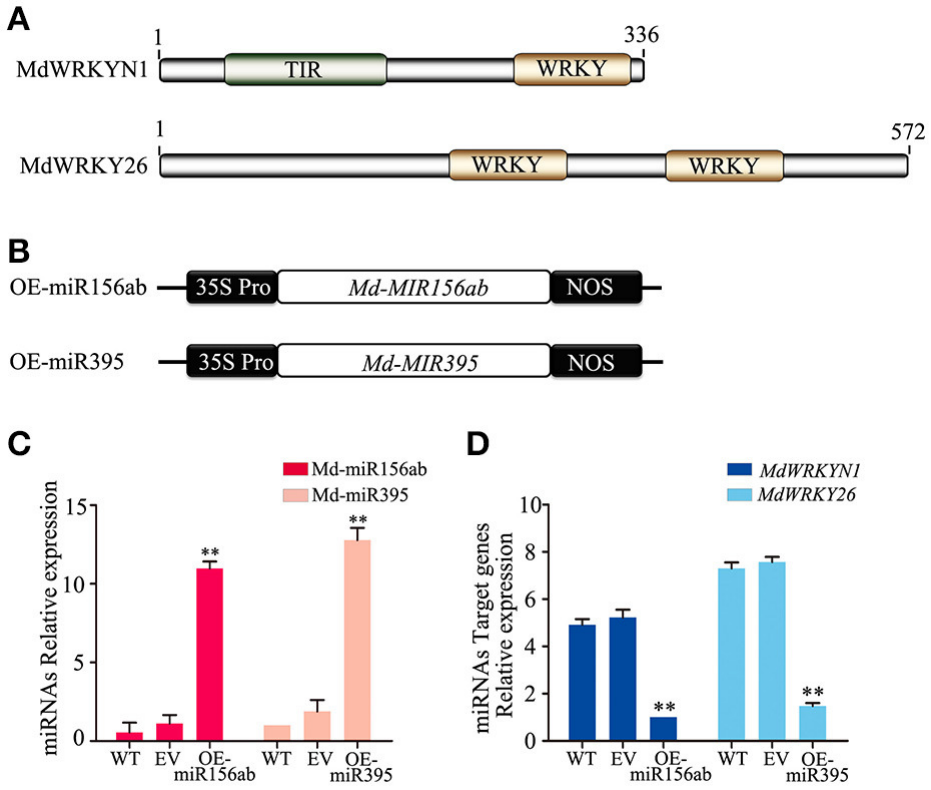
**The miRNAs Md-miR156ab and Md-miR395 are highly induced in ALT1-inoculated “Golden Delicious” (GD) apple leaves, and target WRKY transcription factors. (A)** MdWRKYN1 contains a Toll-like (TIR) domain and WRKY domain. MdWRKY26 contains two WRKY domains. **(B)** Schematic of constructs used for the overexpression (OE-miR156ab/OE-miR395) of miRNAs. **(C)** Real-time PCR confirming the overexpression of Md-miR156ab and Md-miR395 in OE-miR156ab/OE-miR395-infiltrated GD leaves. WT, non-infiltrated GD plants; EV, empty-vector (pFGC5941)-infiltrated plants; OE-miR156ab, Md-miR156ab-overexpressing GD plants; OE-miR395, Md-miR395-overexpressing GD plants. Real-time PCR data were calculated based on three biological and three technical replicates. Error bars = SD; ^**^P < 0.01 (Student's *t*-test). **(D)** Real-time PCR showing the mRNA levels of *MdWRKYN1* and *MdWRKY26* after the infiltration of OE-miR156ab and OE-miR395 in GD, respectively. WT, non-infiltrated GD plants; EV, empty-vector (pFGC5941)-infiltrated plants; OE-miR156ab, Md-miR156ab-overexpressing GD plants; OE-miR395, Md-miR395-overexpressing GD plants. Real-time PCR data were calculated based on three biological and three technical replicates. Error bars = SD; ^**^P < 0.01 (Student's *t*-test).

Notably, except for the apple miRNAs/miRNA families mentioned above (Md-miR156, Md-miR164, Md-miR166, Md-miR159, and Md-miR396) that target five transcription factor families, Md-miR395 (miRBase MIMAT0025980) and Md-miR156ab (miRBase MIMAT0025894) exhibited the largest fold change in abundance after ALT1 infection (Figure 2). Interestingly, Md-miR395 and Md-miR156ab both exhibit the characteristic fold-back RNA secondary structure (Supplemental Figures 4A,B) and target genes encoding WRKY transcription factors (Figure 3, Supplemental Figure 3). Md-miR156ab, which had a 13.69-fold expression difference after ALT1 infection, targets an atypical novel WRKY transcription factor, *MdWRKYN1* (NCBI XM_008353805.1), which contains a Toll/interleukin-1 receptor/resistance protein (TIR) domain as well as a WRKY domain (Figures 2, 3, 4A). Md-miR395 had a 4.98-fold upregulation following ALT1 infection, and regulates a typical WRKY transcription factor, *MdWRKY26* (NCBI XM_008386494.2), containing two WRKY domains (Figures 2, 3, 4A). WRKY transcription factors are key regulatory components of plant responses to biotic and abiotic stresses. Generally, the expression of *WRKY* genes is significantly upregulated following pathogen infection and treatment with salt, drought, and salicylic acid (van Verk et al., 2008; Peng et al., 2012; Choi et al., 2015).

### MdWRKYN1 and MdWRKY26 are suppressed by Md-miR156ab and Md-miR395, respectively

To validate the reliability of the high-throughput sequencing, we analyzed the expression of specific miRNAs targeting transcription factors. Overall, the expression trends of these selected miRNAs determined by real-time PCR were consistent with the sequencing results (Figure 3A), indicating the high reliability of the analysis. To validate whether the predicted target genes were regulated by miRNAs under ALT1 infection, we conducted an expression analysis of the predicted target genes and their corresponding miRNAs. The predicted target genes were downregulated during ALT1 infection, which coincided with an upregulation of their corresponding miRNAs (Figure 3B).

The expression patterns and complementary sequences of Md-miR156ab and *MdWRKYN1* suggest that Md-miR156ab is a suppressor of MdWRKYN1 protein biosynthesis. We overexpressed *Md-MIR156ab* by transforming the plant with a constructed binary vector (pFGC5941) containing the Md-miR156ab primary transcript (Figures 4B,C). Md-miR156ab abundance was significantly increased in these apple lines (Figure 4D), which led to a significant downregulation in the levels of *MdWRKYN1* mRNA (Figure 4D). This analysis was also performed using a vector containing *Md-MIR395* (Figure 4B). Md-miR395 expression was significantly upregulated in these transformed lines (Figure 4C), and the levels of its target mRNA *MdWRKY26* were significantly downregulated (Figure 4D).

The WRKY transcription factors bind to W-box domains (TTGACC/T) in the promoters of their target genes, including PR proteins; thus, we focused on 12 *PR* genes with a W box in their promoter whose expression in GD was altered in response to ALT1 infection (Supplemental Figure 5; van Verk et al., 2008; Peng et al., 2012; Choi et al., 2015). The *PATHOGENESIS-RELATED PROTEINS 1* (*MdPR1*; NCBI NM_001311210.1) and *MdPR5* (NCBI XM_008380268.2) genes contained one W box in their promoters; *GLUTATHIONE PEROXIDASE 7* (*MdGPX7*; NCBI XM_008349268.2), *L-ASCORBATE PEROXIDASE 3* (*MdAPX-2*; NCBI XM_008354210.2), *MAJOR ALLERGEN MAL D 1-LIKE 1* (*MdPR10-1*; NCBI XM_008352950.2), and *MdPR10-2* (NCBI NM_001294363.1) contained two W-box sites in their promoters; *ENDOCHITINASE-LIKE* (*MdPR3-1*; NCBI XM_008395323.1), *ACIDIC ENDOCHITINASE-LIKE* (*MdPR3-2*; NCBI XM_008382187.2), *MdPR8* (NCBI AM600694.1), and *MdAPX-1* (NCBI XM_008387034.1) harbored three W-box sites in their promoters; while *MdPR2* (NCBI AM600693.1) and *CLASS II CHITINASE* (*MdPR4;* NCBI AF494397.1) promoters contained four WRKY binding sites (Supplemental Figure 5).

### Six PR proteins may be induced by MdWRKYN1 to enhance resistance in apple

To investigate the function of two WRKY transcription factors, the relationships between Md-miR156ab-regulated *MdWRKYN1* and Md-miR395-regulated *MdWRKY26* and *PR* genes were investigated during ALT1 infection in apple.

**Figure 5:**
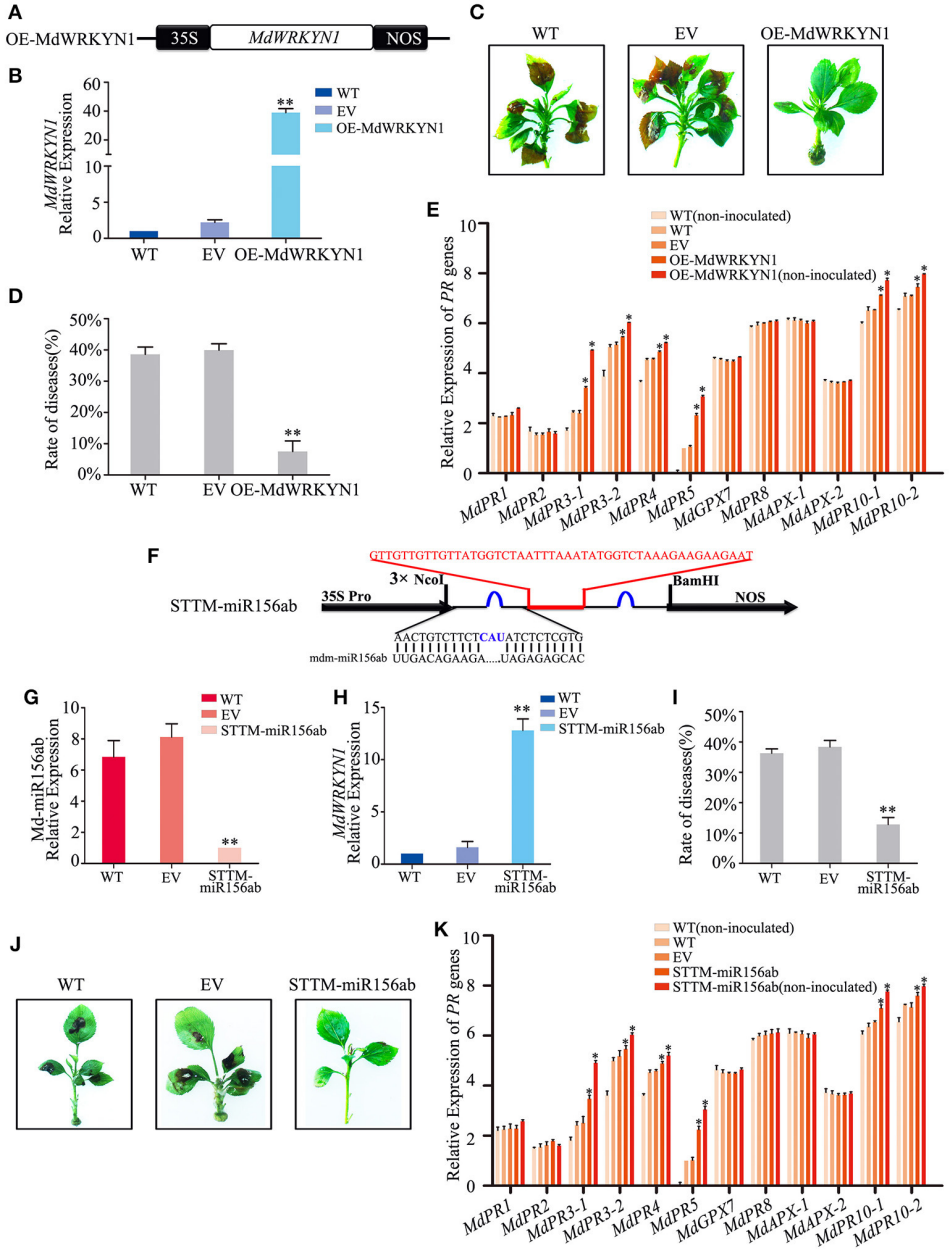
**Md-miR156ab affects ALT1 susceptibility by suppressing the expression of ***MdWRKYN1***, which regulates various ***PR*** genes. (A)** Schematic of constructs used for overexpressing *MdWRKYN1* via *Agrobacterium tumefaciens*-mediated transient expression. **(B)** *MdWRKYN1* content at 24 hpi with ALT1, as revealed by real-time PCR. **(C)** The infection symptoms of WT, EV, and OE-*MdWRKYN1*-infiltrated Golden Delicious (GD) plants. **(D)** Disease rates in GD leaves overexpressing *MdWRKYN1* at 24 hpi with ALT1. **(E)** Expression levels of 12 *PR* genes in GD leaves overexpressing *MdWRKYN1* with or without ALT1 inoculation. In **(B–E)** WT, non-infiltrated GD plants; EV, empty-vector (pFGC5941)-infiltrated plants; OE-*MdWRKYN1, MdWRKYN1*-overexpressing GD plants. Spore inoculum concentration: 2 × 10^5^ CFU/ml. Spore growth was measured at 6 dpi. Error bars = SD; ^**^P < 0.01; ^*^P < 0.05 (Student's *t*-test). Real-time PCR data were calculated based on three biological and three technical replicates. **(F)** Schematic of Short Tandem Target Mimic (STTM) constructs used for silencing Md-miR156ab via *Agrobacterium tumefaciens*-mediated transient expression. Red indicates the spacer region and the spacer sequence. Blue indicates the bulge sequences in the miRNA binding sites. **(G)** Real-time PCR confirming knockdown of Md-miR156ab activity. **(H)** *MdWRKYN1* expression 24 hpi with ALT1, as revealed by real-time PCR analysis. **(I)** Disease rate in GD leaves following the reduction in Md-miR156ab activity at 24 hpi with ALT1. **(J)** Infection symptoms of STTM-miR156ab-infiltrated ALT1-inoculated GD plants in comparison with wild-type and empty-vector controls. **(K)** Expression of 12 *PR* genes in GD leaves in STTM-miR156ab-infiltrated plants, along with wild-type and empty-vector controls, at 24 hpi with ALT1. In **(G–K)** WT, non-infiltrated GD plants; EV, empty-vector (pFGC5941)-infiltrated plants; STTM-miR156ab, GD plants with silenced Md-miR156ab activity. Spore inoculum concentration: 2 × 10^5^ CFU/ml. Spore growth was measured at 6 dpi. Error bars = SD; ^**^P < 0.01; ^*^P < 0.05 (Student's *t*-test). Real-time PCR data were calculated based on three biological and three technical replicates.

GD leaves were transformed to overexpress *MdWRKYN1* (Figure 5A). Four days after infiltration, *MdWRKYN1* expression was found to be significantly upregulated in these plants (Figure 5B). After ALT1 inoculation, the disease rate in the *MdWRKYN1*-overexpressing plants (7.50%) was significantly lower than in the wild-type (WT; 38.59%) and empty-vector (EV; 39.96%) plants, and the symptoms of ALT1 infection were suppressed. Resistance to apple leaf spot disease was thus increased in *MdWRKYN1*-overexpressing plants (Figures 5C,D, Supplemental Table 5). Real-time PCR showed that the expression levels of *MdPR3-1, MdPR3-2, MdPR4, MdPR5, MdPR10-1*, and *MdPR10-2* were increased in OE-*MdWRKYN1* plants after ALT1 inoculation compared with the controls (Figure 5E). Overexpressing *MdWRKYN1* in the absence of ALT1 inoculation confirmed that the six upregulated *PR* genes were induced by the MdWRKYN1 transcription factor (Figure 5E). These results suggest that ALT1 infection induces Md-miR156ab expression, which suppresses *MdWRKYN1* transcription. Overexpressing *MdWRKYN1* enhances the disease resistance of GD through activation of *MdPR3-1, MdPR3-2, MdPR4, MdPR5, MdPR10-1*, and *MdPR10-2* expression.

We synthesized a Short Tandem Target Mimic (STTM) sequence to block the target sites of Md-miR156ab, fused it into the vector pFGC5941 (Figure 5F), and transformed it into GD apple (Tang et al., 2012; Yan et al., 2012; Tang and Tang, 2013; Reichel et al., 2015). After 4 days, real-time PCR indicated a significant decrease in the abundance of Md-miR156ab expression (Figure 5G). The STTM-miR156ab plants were inoculated with ALT1, and at 24 hpi these plants had a significant increase in *MdWRKYN1* expression (Figure 5H), further confirming Md-miR156ab as a regulator of *MdWRKYN1* expression in apple. The STTM-miR156ab-expressing plants had a significantly lower rate of disease (12.83%) than the WT (36.26%) or EV (38.40%) plants, and showed a greater resistance to apple leaf spot disease (Figures 5I,J, Supplemental Table 6). Real-time PCR results showed that *MdPR3-1, MdPR3-2, MdPR4, MdPR5, MdPR10-1*, and *MdPR10-2* expression levels were significantly higher in the STTM-miR156ab-expressing plants following ALT1 inoculation than in the wild-type and empty-vector controls (Figure 5K). Thus, we concluded that resistance to apple leaf spot disease is under the influence of the *PR* genes, whose accumulation is regulated by *MdWRKYN1*, which is suppressed by Md-miR156ab.

### MdWRKY26 may induce eight *PR* genes to regulate apple resistance

**Figure 6:**
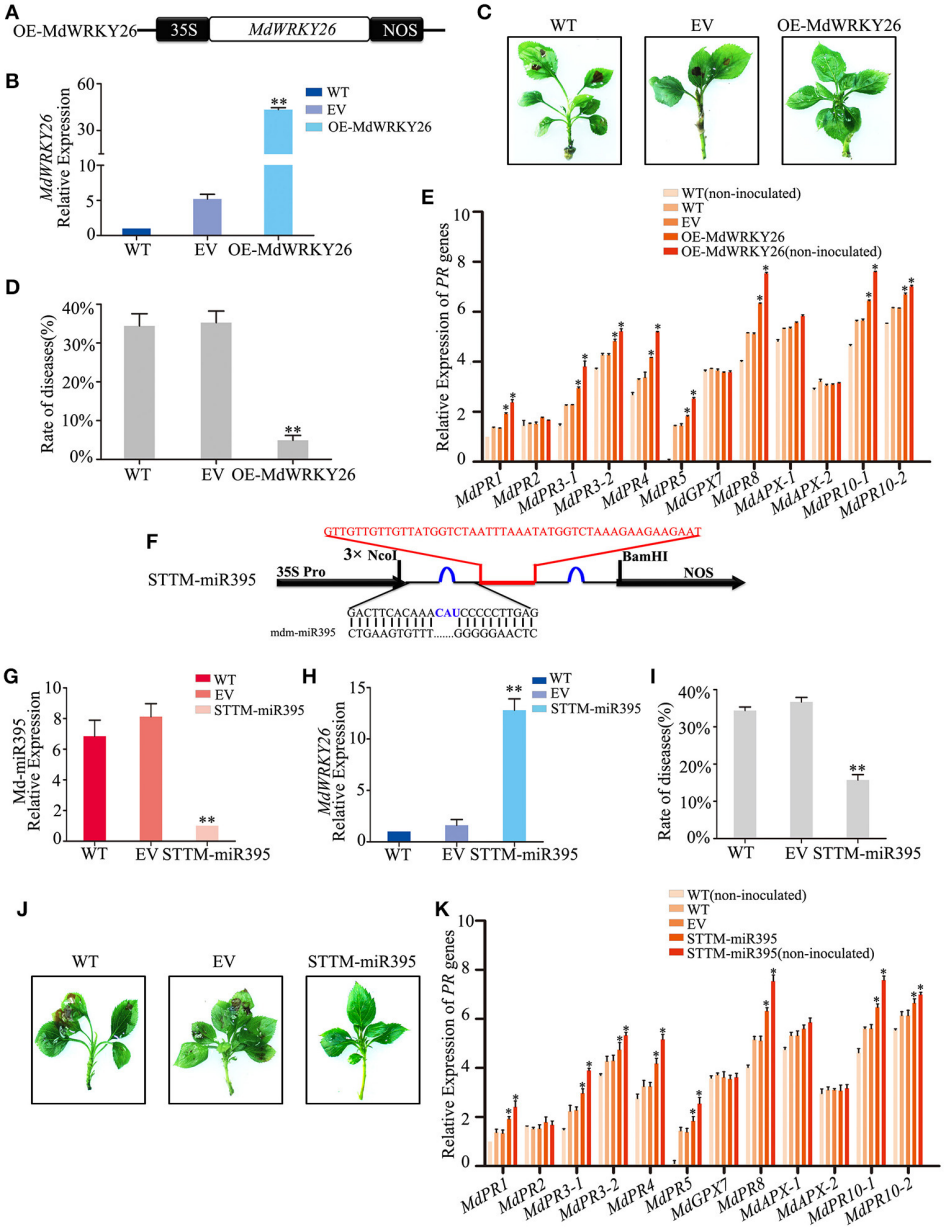
**Md-miR395 affects ALT1susceptibility by suppressing the expression of ***MdWRKY26***, which regulates various ***PR*** genes. (A)** Schematic of constructs used for overexpressing *MdWRKY26* via *Agrobacterium tumefaciens*-mediated transient expression. **(B)** *MdWRKY26* content at 24 hpi with ALT1, as revealed by real-time PCR. **(C)** The infection symptoms of WT, EV, and OE-*MdWRKY26*-infiltrated Golden Delicious (GD) plants. **(D)** Disease rates in GD leaves overexpressing MdWRKY26 at 24 hpi with ALT1. **(E)** Expression levels of 12 *PR* genes in GD leaves overexpressing *MdWRKY26* with or without ALT1 inoculation. In **(B–E)** WT, non-infiltrated GD plants; EV, empty-vector (pFGC5941)-infiltrated plants; OE-*MdWRKY26, MdWRKY26*-overexpressing GD plants. Spore inoculum concentration: 2 × 10^5^ CFU/ml. Spore growth was measured at 6 dpi. Error bars = SD; ^**^P < 0.01; ^*^P < 0.05 (Student's *t*-test). Real-time PCR data were calculated based on three biological and three technical replicates. **(F)** Schematic of Short Tandem Target Mimic (STTM) constructs used for silencing Md-miR395 via *Agrobacterium tumefaciens*-mediated transient expression. **(G)** Real-time PCR confirming the knockdown of Md-miR395 activity. **(H)** *MdWRKY26* expression at 24 hpi with ALT1, as revealed by real-time PCR analysis. **(I)** Disease rates in GD leaves absorbing Md-miR395 at 24 hpi with ALT1. **(J)** The infection symptoms of STTM-miR395-infiltrated ALT1-inoculated GD plants in comparison with wild-type and empty-vector controls. **(K)** Expression of 12 *PR* genes in GD leaves in STTM-Md-miR395-infiltrated plants, along with wild-type and empty-vector controls at 24 hpi with ALT1. In **(G–K)** WT, non-infiltrated GD plants; EV, empty-vector (pFGC5941)-infiltrated plants; STTM-miR395, GD plants with silenced Md-miR395 activity. Spore inoculum concentration: 2 × 10^5^ CFU/ml. Spore growth was measured at 6 dpi. Error bars = SD; ^**^P < 0.01; ^*^P < 0.05 (Student's *t*-test). Real-time PCR data were calculated based on three biological and three technical replicates.

To investigate whether MdWRKY26, which contains two WRKY domains, could play a role in plant defense against apple leaf spot disease, we overexpressed *MdWRKY26* and silenced its regulatory miRNA, Md-miR395. We constructed a binary vector (pFGC5941) containing the coding sequence of *MdWRKY26* (Figure 6A), then transformed it into the susceptible apple variety GD. Four days after transformation, *MdWRKY26* expression was significantly upregulated (Figure 6B). The transformed leaves were inoculated with ALT1; at 24 hpi, 34.41% of the WT plants and 35.26% of the EV plants showed symptoms of leaf spot disease, while only 4.92% of the OE-*MdWRKY26* plants showed susceptibility to ALT1 (Figures 6C,D, Supplemental Table 7). Real-time PCR analysis showed that *MdPR1, MdPR3-1, MdPR3-2, MdPR4, MdPR5, MdPR8, MdPR10-1*, and *MdPR10-2* expression increased in OE-*MdWRKY26* plants either with or without ALT1 inoculation, relative to the wild-type and empty-vector controls (Figure 6E). These results suggest that ALT1 infection induces the production of Md-miR395, which suppresses *MdWRKY26*, while the overexpression of *MdWRKY26* enhances the disease resistance of GD through the induction of *MdPR1, MdPR3-1, MdPR3-2, MdPR4, MdPR5, MdPR8, MdPR10-1*, and *MdPR10-2* expression.

We synthesized a STTM sequence to block the binding of Md-miR395 to its target sequences, fused it into the vector pFGC5941 (Figure 6F), and expressed STTM-miR395 in the susceptible apple variety GD. After 4 days, real-time PCR indicated a significant decrease in Md-miR395 transcripts (Figure 6G). At 24 hpi with ALT1, we identified an alleviation of the Md-miR395-induced suppression of *MdWRKY26* transcripts (Figure 6H), further confirming Md-miR395 as a regulator of *MdWRKY26* expression in apple. The STTM-miR395-expressing plants had a lower rate of disease (15.71%) compared to the WT (34.37%) and EV (36.69%) plants, indicating an improved resistance to apple leaf spot disease (Figures 6I,J, Supplemental Table 8). Real-time PCR showed that *MdPR1, MdPR3-1, MdPR3-2, MdPR4, MdPR5, MdPR8, MdPR10-1*, and *MdPR10-2* expression levels were significantly higher in the *STTM-miR395*-expressing plants after ALT1 inoculation than in the wild-type and empty-vector controls (Figure 6K). We therefore preliminarily consider that apple leaf spot disease may be related, in the pathosystem we have investigated, to the *PR* genes, whose accumulation is regulated by *MdWRKY26* in the absence of suppression by Md-miR395.

## Discussion

Apple is considered a model fruit plant due to its high level of production and worldwide economic value; however, to date, apple production has been limited by many kinds of fungal diseases. *Alternaria* blotch, caused by the *Alternaria alternaria f. sp. mali*, has become a destructive apple disease in China and other East Asian countries. The disease causes circular blackish spots on apple leaves in late spring or early summer, resulting in serious defoliation and decreased fruit quality in field (Zhang et al., 2015).

Previous high-throughput analyses of the proteome and transcriptome provided useful insights into the molecular differences between apple leaves in the presence or absence of ALT1 infection (Zhang et al., 2015; Huang et al., 2016); however, there have been no reports of a high-throughput sRNA analysis in apple. A few studies have reported the presence of conserved abiotic- and biotic-stress-induced miRNAs in apple, such as miR164, miR159, and miR171, suggesting that sRNAs also play an important role in defense in this plant (Zhang et al., 2011). Also, Md-miR171 had the greatest difference in expression following ALT1 inoculation. In the present study, next-generation sequencing of miRNAs revealed 39 previously identified and 19 novel miRNAs with over a 2-fold difference in expression following ALT1 infection. This relatively high number of differentially expressed miRNAs may be a reflection of a complex molecular mechanism of response to pathogens.

We identified 78 putative target genes of the miRNAs with differential expression in the presence of the ALT1 pathogen, of which nine were transcription factors. Two of these targets were part of the WRKY family of transcription factors, which have previously been reported to be key factors in plant stress responses (Rushton et al., 2010; Chi et al., 2013; Schluttenhofer and Yuan, 2015; Phukan et al., 2016). The miRNA Md-miR156ab targeted transcripts encoding a WRKY transcription factor, MdWRKYN1, while *MdWRKY26* transcripts were targeted by Md-miR395. WRKY proteins were initially classified into three groups based on the number and structure of the conserved WRKY zinc-finger motifs; the first group contains proteins with two Cx4Cx22–23HxH zinc-finger motifs, while proteins of the second group contain one Cx4–5Cx23HxH zinc-finger motif, and those in the third group contain one Cx7Cx23HxC zinc-finger motif. More recent analyses have shown that WRKY proteins can be further divided into subgroups (Rushton et al., 2010; Chi et al., 2013; Schluttenhofer and Yuan, 2015; Phukan et al., 2016). In the present study, MdWRKY26 was found to be a conserved type of WRKY transcription factor containing a double WRKY domain. MdWRKYN1 was shown to contain a single WRKY domain, but is a novel type of WRKY transcription factor because it also contains a TIR domain (Mohanta et al., 2016). There have been no previous reports of the function of this novel transcription factor; however, it is possible that it could integrate different types of signal via its TIR and WRKY domains, making MdWRKYN1 an interesting subject for further research.

While the expression of *WRKY* genes is known to be induced by various plant stresses (Rushton et al., 2010; Chi et al., 2013; Schluttenhofer and Yuan, 2015; Phukan et al., 2016), few studies have reported on their post-transcriptional regulation. In the present study we show that, while ALT1 inoculation induced the upregulation of Md-miR156ab and Md-miR395 expression, this alone did not result in a dramatic suppression of their target *WRKY* genes, possibly because these genes may also be activated by other factors. When *MdWRKYN1* and *MdWRKY26* were overexpressed, however, disease resistance was improved, leading us to investigate downstream genes regulated by these transcription factors.

Most WRKY transcription factors target a core promoter element, the W box, which is present in the promoters of many PR proteins (van Verk et al., 2008; Peng et al., 2012; Choi et al., 2015). In *Nicotiana tabacum* (tobacco) and rice, WRKY transcription factors activate *PR1* and *PR10* gene expression inducted by salicylic acid and bacterial elicitors (van Verk et al., 2008; Peng et al., 2012; Choi et al., 2015). Until now, however, there have been few reports about the relationship between WRKY transcription factors and PRs in apple. In the present study, we identified 12 *PR* genes in apple with a W box in their promoters, and determined that the expression of some of these genes could be induced by MdWRKYN1 or MdWRKY26. We detected that expression of the PR genes *MdPR3-1, MdPR3-2, MdPR4, MdPR5, MdPR10-1*, and *MdPR10-2* was greater in plants overexpressing *MdWRKYN1* without ALT1 inoculation than in those that had been infected. This may be a result of the higher level of Md-miR156ab expression in the ALT1-inoculated plants, which suppresses MdWRKYN1 production and results in a lower expression of *PR* genes. Plants overexpressing *MdWRKY26* expressed lower levels of *MdPR1, MdPR3-1, MdPR3-2, MdPR4, MdPR5, MdPR8, MdPR10-1*, and *MdPR10-2* when infected with ALT1.

**Figure 7:**
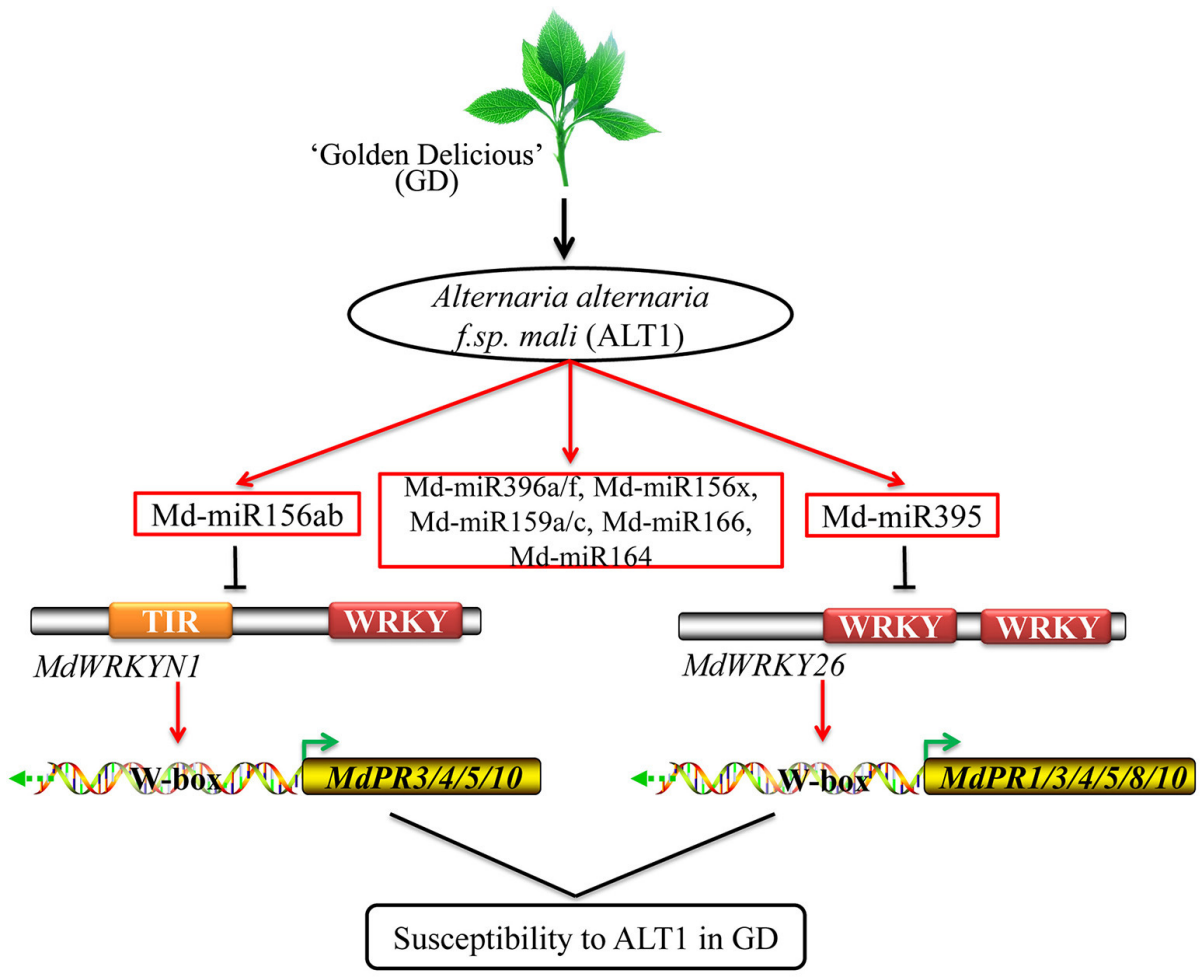
**Model for the role of Md-miR156ab and Md-miR395 in GD plants infected with ALT1**. ALT1-induced Md-miR156ab and Md-miR395 suppress *MdWRKYN1* and *MdWRKY26* expression, thereby decreasing the expression of some PR genes, and resulting in susceptibility to ALT1 in GD.

PR proteins accumulate after infection with pathogens, and may act as anti-fungal agents by performing antimicrobial activities including cell wall hydrolysis and contact toxicity, and perhaps playing a role in defense signaling. *PR1* was first identified in tobacco, and its upregulation has been shown to enhance resistance to fungal and bacterial disease in *Arabidopsis*, rice, *Solanum lycopersicum* (tomato), *Triticum aestivum* (wheat), and apple (Cao et al., 1998; Chern et al., 2001, 2005; Friedrich et al., 2001; Fitzgerald et al., 2004; Lin et al., 2004; Makandar et al., 2006; Malnoy et al., 2007). *PR3* encodes an endochitinase, which could provide protection against fungal pathogens, while a PR4-type protein was shown to possess ribonuclease activity that could protect against the fungal pathogen *Botryosphaeria dothidea* in apple (Bai et al., 2013; Hassani et al., 2016). Some other *PR* genes, including *PR5* and *PR8*, were also reported to enhance resistance in apple, while PR8 and related proteins possess lysozyme activity and may be directed against bacteria (Hassani et al., 2016). Some PR10 proteins show homology to ribonuclease, but in apple, *PR10* expression can be induced by pathogens, suggesting that it may participate in plant defense (Puehringer et al., 2003; Nucera et al., 2010). In GD, ALT1-induced Md-miR156ab and Md-miR395 suppress *MdWRKYN1* and *MdWRKY26* expression then decrease *MdPR1, MdPR3-1, MdPR3-2, MdPR4, MdPR5, MdPR8, MdPR10-1*, and *MdPR10-2* expression, resulting in susceptibility to ALT1. These findings provide a theoretical basis for deciphering the mechanisms regulating susceptibility to apple leaf spot disease in GD (Figure 7).

Molecular mechanisms are more readily deduced using tissue-cultured plants, as they can be grown at a controlled temperature, humidity, and photoperiod. Besides experimental inoculations, the cultured plants will not be exposed to pathogens, which remove confounding variables. Tissue-cultured apple plants are usually derived from explants of apple trees growing in the field (Belaizi et al., 1991), and may therefore have similar molecular mechanisms to field-grown plants. For this reason, we propose that the susceptibility mechanisms that we have elucidated here using tissue cultures could be reflected in GD apple trees in the field. The progenitors of the domesticated apple came from crosses between the wild apple of central Asia and its close relatives (Harris et al., 2002), which likely shared similar defense mechanisms. Apple cultivars that are closely related to GD may therefore share a similar susceptibility mechanism in response to ALT1.

Based on our study, ALT1-induced Md-miR156ab and Md-miR395 suppress *MdWRKYN1* and *MdWRKY26* expression, thereby decreasing the expression of some *PR* genes, and resulting in susceptibility to ALT1 in GD (Figure 7). In resistant cultivars, we speculate that ALT1 may not be able to induce the production of Md-miR156ab and Md-miR395, or may somehow induce *MdWRKYN1* and *MdWRKY26* expression, increasing the expression of the *PR* genes and resulting in resistance to this pathogen. In the future, we would like to investigate the mechanism of apple leaf spot defense in resistant cultivars and elucidate the cultivar-specific differences that result in resistance or susceptibility to ALT1.

## Author contributions

QZ and YL: designed experiments; QZ, YZ, CW, and SW: carried out experiments; QZ, LH, and SW: analyzed experimental results; QZ and TL: wrote the manuscript.

### Conflict of interest statement

The authors declare that the research was conducted in the absence of any commercial or financial relationships that could be construed as a potential conflict of interest.

## Supplementary material

Supplemental Figure 1 **Analysis of the base preferences of previously identified small RNAs. (A)** Distribution of the first nucleotides in previously identified small RNAs. **(B)** Distribution of nucleotides throughout the length of previously identified small RNAs.

Supplemental Figure 2 **Distribution of confirmed miRNA targets separated by category. (A)** Classification of miRNA targets according to GO. **(B)** Classification of miRNA targets according to KEGG.

Supplemental Figure 3 **The complementation of miRNAs with their target transcription factors**.

Supplemental Figure 4 **The characteristic fold-back RNA secondary structure of Md-miR395 and Md-miR156ab. (A)** The stem-loop structure of Md-miR156ab. **(B)** The stem-loop structure of Md-miR395. The red line indicates the position and sequence of Md-miR156ab and Md-miR395.

Supplemental Figure 5 **The number of W-box domains on the promoters of ***PR*** genes**.

Supplemental Table 1 **Sequences and accession numbers of the 39 differentially expressed previously identified mature miRNAs**. The accession numbers were obtained from miRBase (http://www.mirbase.org/).

Supplemental Table 2 **Sequences of the 19 differentially expressed novel mature miRNAs and their precursors**.

Supplemental Table 3 **Primer sequences used to clone the mature miRNAs**.

Supplemental Table 4 **Primer sequences used to perform real time-PCR**.

Supplemental Table 5 **The disease rate of WT, EV, and OE-***MdWRKYN1*** and ***t***-test analysis**. Independent *t*-test demonstrated that there was significant difference (*P* < 0.01) in the disease rate of EV and OE-*MdWRKYN1* under ALT1-inoculated. WT, non-infiltrated GD plants; EV, empty-vector (pFGC5941)-infiltrated plants; OE-*MdWRKYN1, MdWRKYN1*-overexpressing GD plants.

Supplemental Table 6 **The disease rate of WT, EV, and STTM-miR156ab and ***t***-test analysis**. Independent *t*-test demonstrated that there was significant difference (*P* < 0.01) in the disease rate of EV and STTM-miR156ab under ALT1-inoculated. WT, non-infiltrated GD plants; EV, empty-vector (pFGC5941)-infiltrated plants; STTM-miR156ab, GD plants with silenced Md-miR156ab activity.

Supplemental Table 7 **The disease rate of WT, EV, and OE-***MdWRKYN26*** and ***t***-test analysis**. Independent *t*-test demonstrated that there was significant difference (*P* < 0.01) in the disease rate of EV and OE-*MdWRKYN26* under ALT1-inoculated. WT, non-infiltrated GD plants; EV, empty-vector (pFGC5941)-infiltrated plants; OE-*MdWRKY26, MdWRKY26*-overexpressing GD plants.

Supplemental Table 8 **The disease rate of WT, EV, and STTM-miR395 and ***t***-test analysis**. Independent *t*-test demonstrated that there was significant difference (*P* < 0.01) in the disease rate of EV and STTM-miR395 under ALT1-inoculated. WT, non-infiltrated GD plants; EV, empty-vector (pFGC5941)-infiltrated plants; STTM-miR395, GD plants with silenced Md-miR395 activity.
