# Supplementary figures and images for: Md-miR156ab and Md-miR395 Target WRKY Transcription Factors to Influence Apple Resistance to Leaf Spot Disease
Source: Front Plant Sci. 2017 Apr 19;8:526. doi: 10.3389/fpls.2017.00526 (PMC5395612; doi:10.3389/fpls.2017.00526)

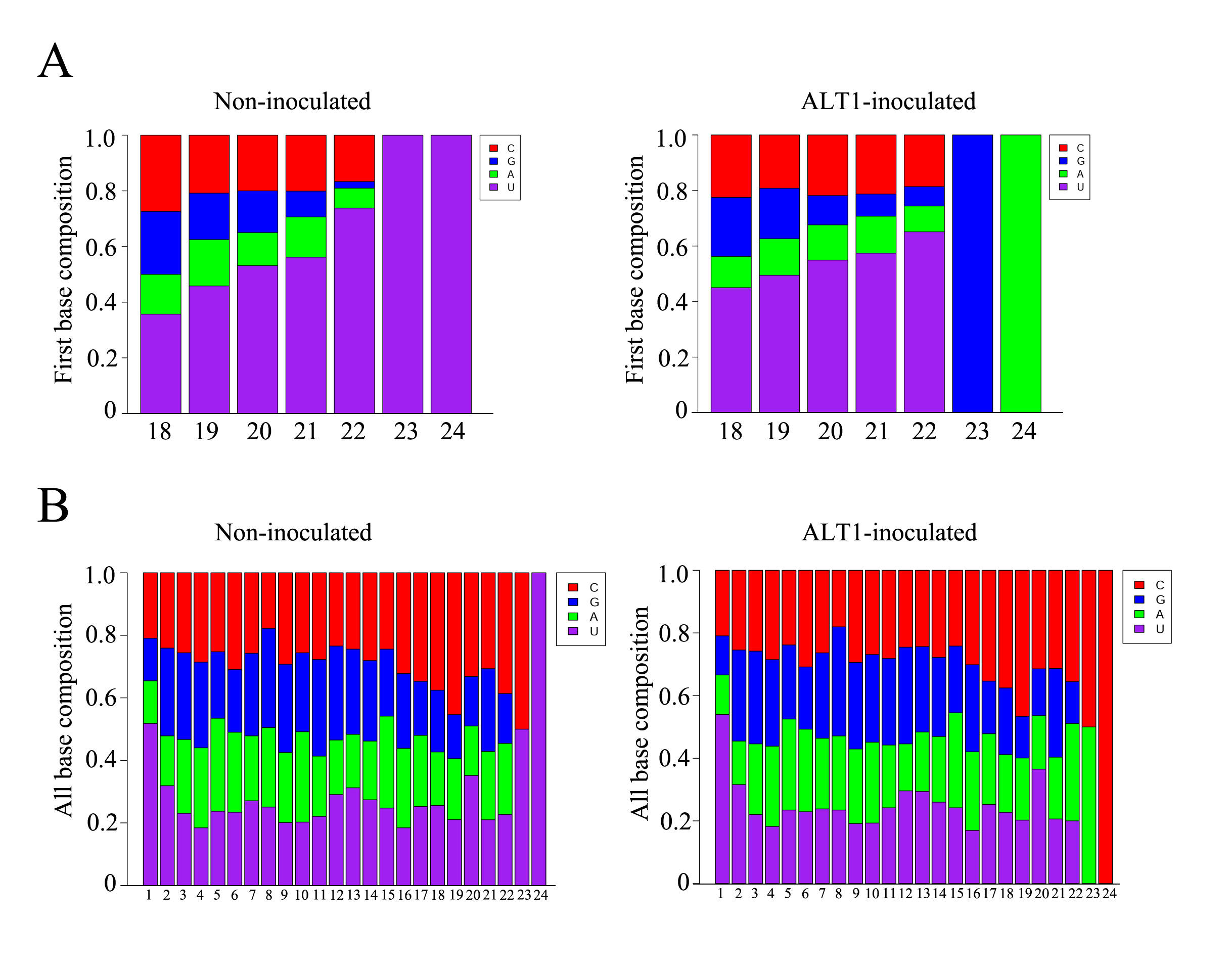

Supplement: Supplemental Figure 1 — Analysis of the base preferences of previously identified small RNAs. (A) Distribution of the first nucleotides in previously identified small RNAs. (B) Distribution of nucleotides throughout the length of previously identified small RNAs. [file Image1.JPEG]

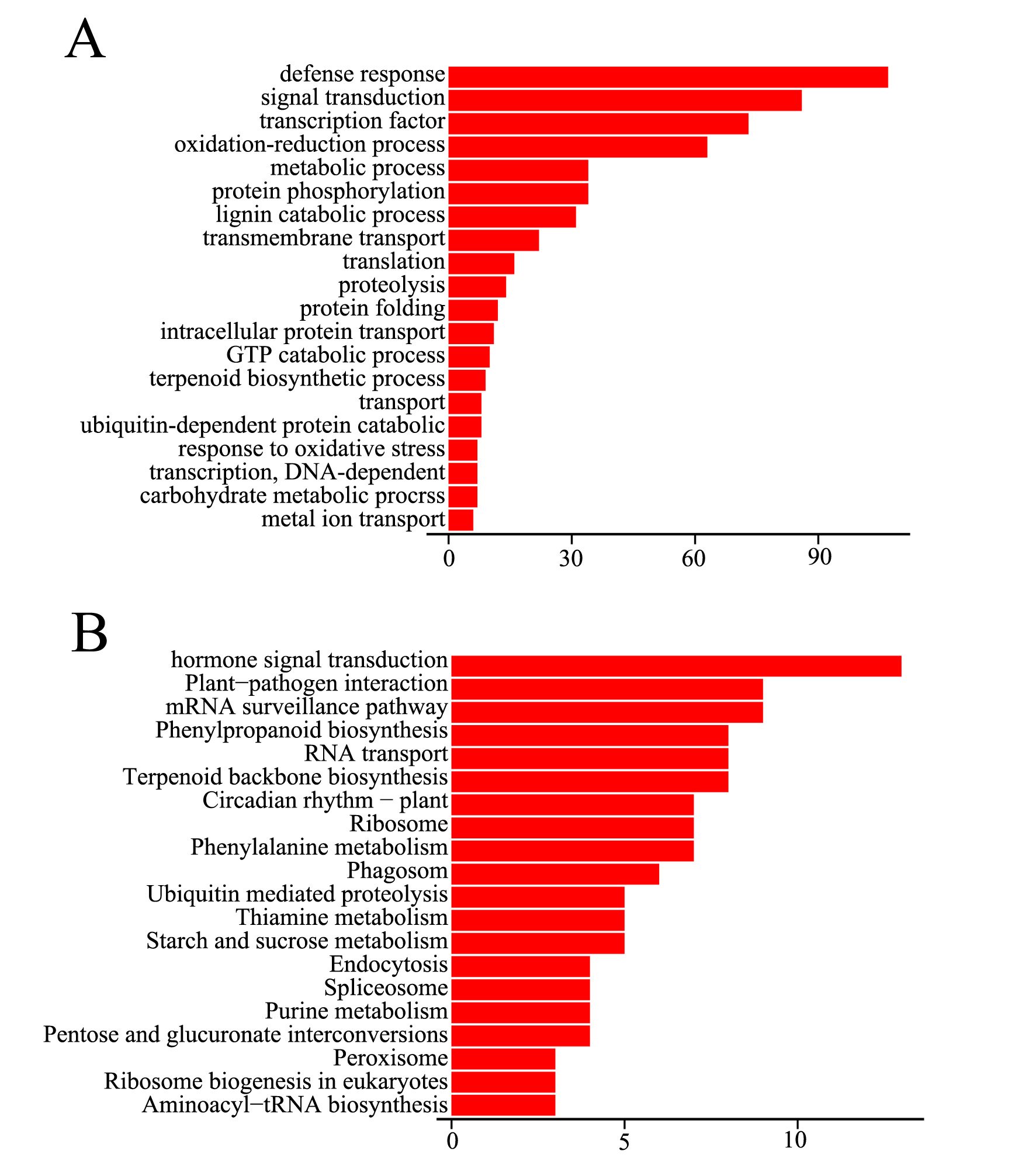

Supplement: Supplemental Figure 2 — Distribution of confirmed miRNA targets separated by category. (A) Classification of miRNA targets according to GO. (B) Classification of miRNA targets according to KEGG. [file Image2.JPEG]

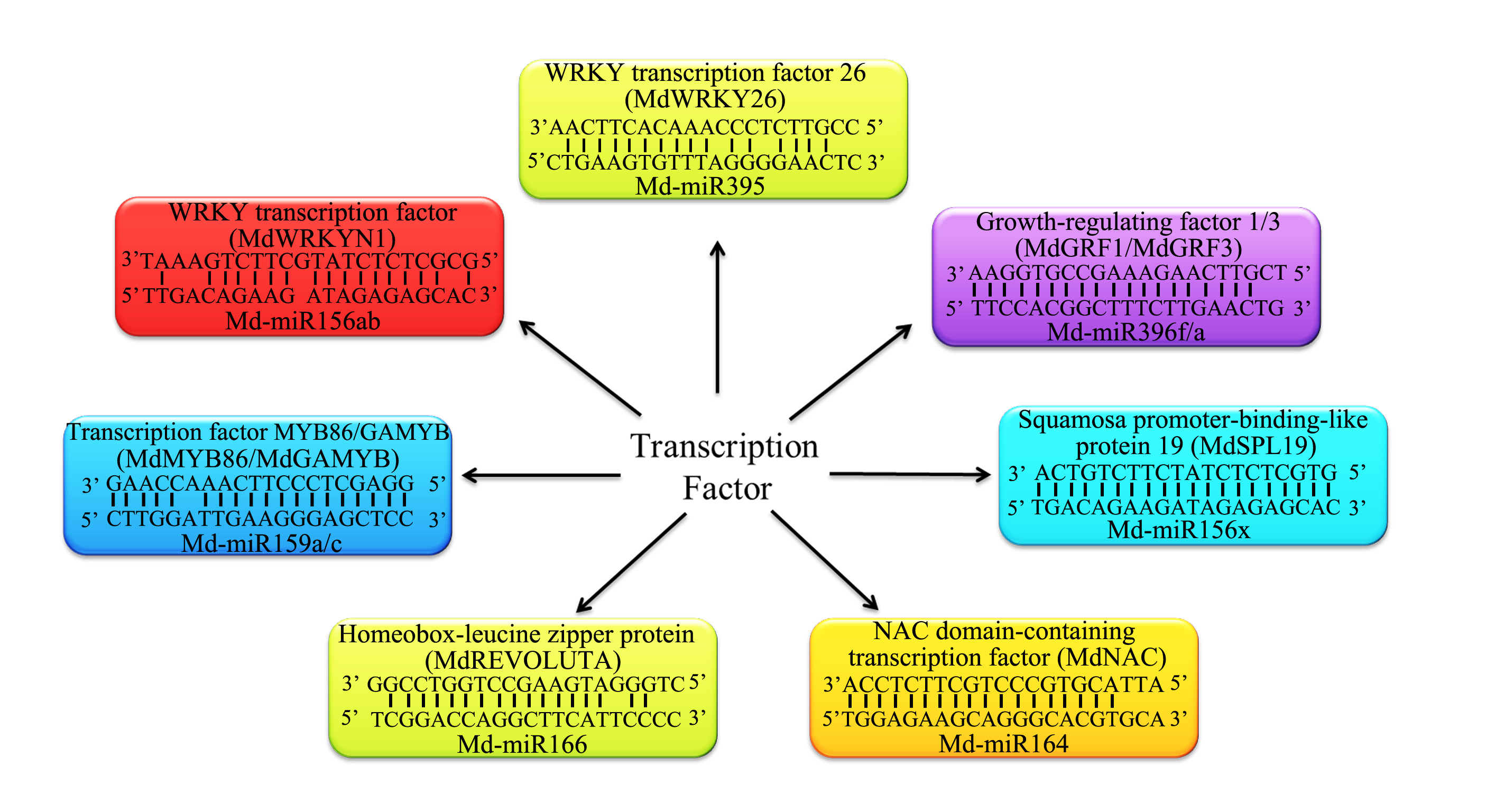

Supplement: Supplemental Figure 3 — The complementation of miRNAs with their target transcription factors. [file Image3.JPEG]

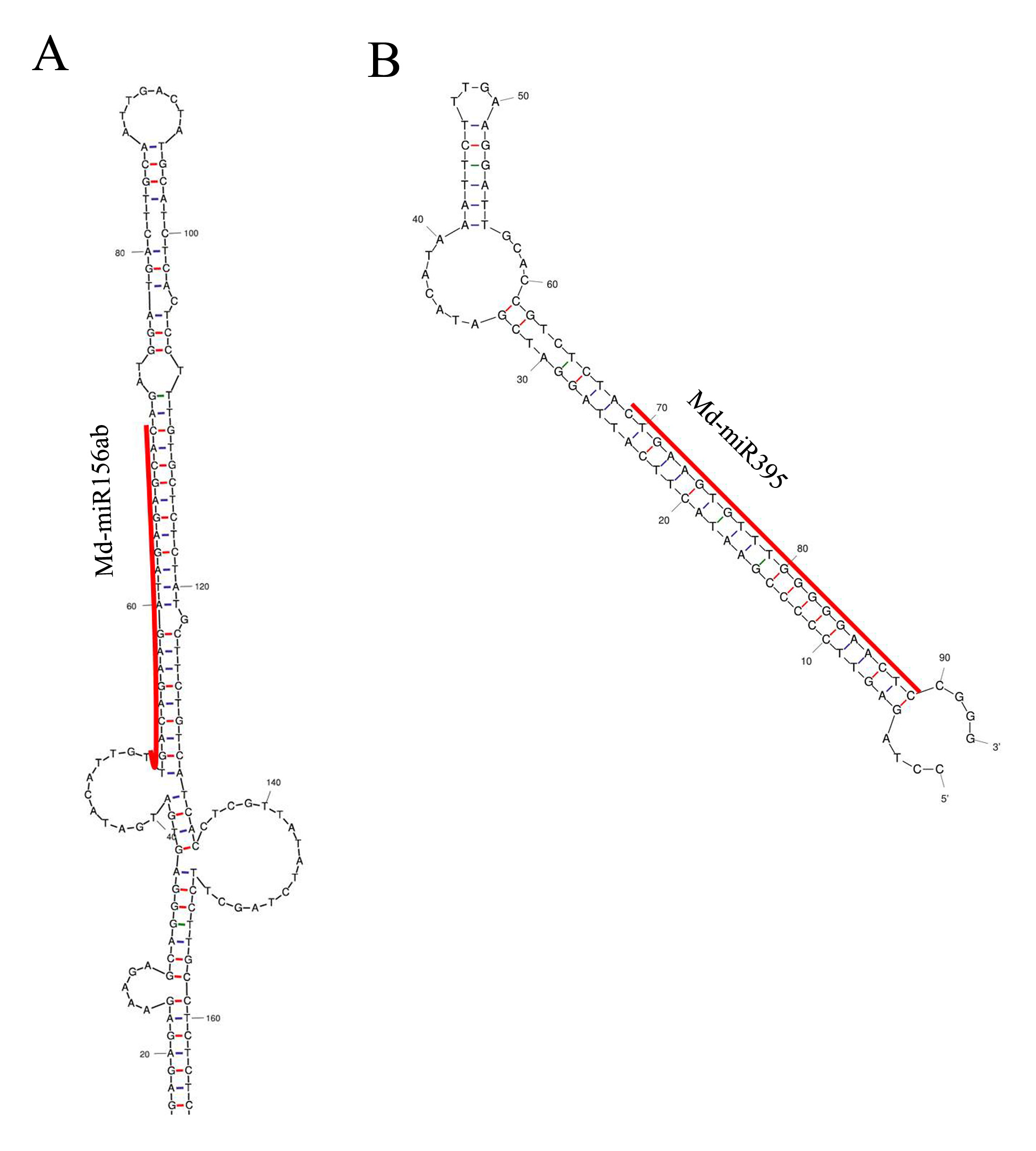

Supplement: Supplemental Figure 4 — The characteristic fold-back RNA secondary structure of Md-miR395 and Md-miR156ab. (A) The stem-loop structure of Md-miR156ab. (B) The stem-loop structure of Md-miR395. The red line indicates the position and sequence of Md-miR156ab and Md-miR395. [file Image4.JPEG]

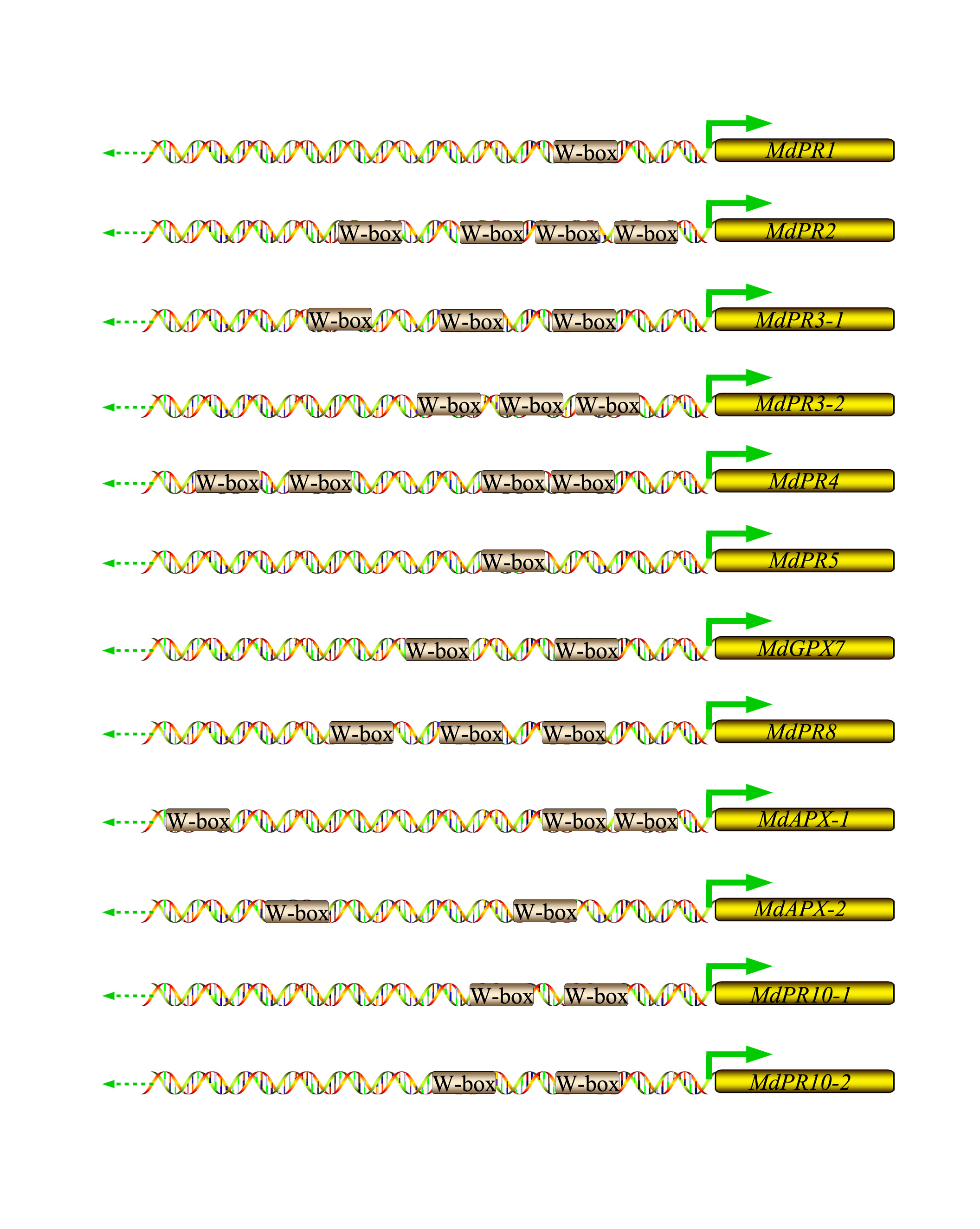

Supplement: Supplemental Figure 5 — The number of W-box domains on the promoters of PR genes. [file Image5.JPEG]
