# Supplementary material for: Md-miR156ab and Md-miR395 Target WRKY Transcription Factors to Influence Apple Resistance to Leaf Spot Disease
Source: Front Plant Sci. 2017 Apr 19;8:526. doi: 10.3389/fpls.2017.00526 (PMC5395612; doi:10.3389/fpls.2017.00526)
Supplement: Supplemental Table 1 — Sequences and accession numbers of the 39 differentially expressed previously identified mature miRNAs. The accession numbers were obtained from miRBase (http://www.mirbase.org/). [file Table1.PDF]

Supplemental Table 1. The sequences of the 39 differently expressed previously identified mature miRNAs.

Previously identified miRNAs	Sequence	Accession number
Md-miR171g	TGATTGAGCCGTGCCAATATC	MIMAT0025944
Md-miR171a	TTGAGCCGCGTCAATATCTCC	MIMAT0025938
Md-miR398a	TGTGTTCTCAGGTCACCCCTT	MIMAT0025998
Md-miR156ab	TTGACAGAAGATAGAGAGCAC	MIMAT0025894
Md-miR7121g	TCCTCTTGGTGATCGCCCTGC	MIMAT0026046
Md-miR156e	TGACAGAAGAGAGTGAGCAC	MIMAT0025871
Md-miR156p	CTGACAGAAGATAGAGAGCAC	MIMAT0025882
Md-miR393	TCCAAAGGGATCGCATTGATCT	MIMAT0025975
Md-miR395	CTGAAGTGTTTGGGGGAACTC	MIMAT0025980
Md-miR482a-5p	AGGAATGGGCTGTTTGGGAAGA	MIMAT0011164
Md-miR2111	TCCTTGGGATGCAGATTACCT	MIMAT0026014
Md-miR396f	TTCCACGGCTTTCTTGAACTG	MIMAT0025994
Md-miR858	TTCGTTGTCTGTTCGACCTGA	MIMAT0026070
Md-miR168a	TCGCTTGGTGCAGGTCGGGAA	MIMAT0025932
Md-miR156x	TGACAGAAGATAGAGAGCAC	MIMAT0025890
Md-miR7122	TTATACAGAGAAATCACGGTCG	MIMAT0026048
Md-miR162	TCGATAAACCTCTGCATCCAG	MIMAT0025905
Md-miR403	TTAGATTCACGCACAAACTCG	MIMAT0026011
Md-miR7123	AAGAGCGGGATGTGTAAAAGG	MIMAT0026050
Md-miR397	TTGAGTGCAGCGTTGATGAAA	MIMAT0025996
Md-miR167h	TGAAGCTGCCAGCATGATCTTA	MIMAT0025929
Md-miR5225	TCTGTCGTGGGTGAGATGGTGC	MIMAT0026052
Md-miR168b	TGGTGCAGGTCGGGAACCGCT	MIMAT0025933
Md-miR164	TGGAGAAGCAGGGCACGTGCA	MIMAT0025908
Md-miR482a-3p	TTCCCAAGCCCGCCCATTCCTA	MIMAT0011165
Md-miR159a	CTTGGATTGAAGGGAGCTCC	MIMAT0025898
Md-miR159c	GAATTCCTTCTCCTCTCCTTT	MIMAT0026053
Md-miR156t	TTGACAGAAGAGAGAGAGCAC	MIMAT0025886
Md-miR156a	TGACAGAAGAGAGTGAGCAC	MIMAT0025867
Md-miR535	TGACGACGAGAGAGAGCACGC	MIMAT0026027
Md-miR171f	TTGAGCCGTGCCAATATCACG	MIMAT0025943
Md-miR3627	TCGCAGGAGAGATGGCACTA	MIMAT0026016
Md-miR396a	TTCCACAGCTTTCTTGAACAG	MIMAT0025989
Md-miR166	TCGGACCAGGCTTCATTCCCC	MIMAT0025913
Md-miR167b	TGAAGCTGCCAGCATGATCTA	MIMAT0025923
Md-miR319	TTGGACTGAAGGGAGCTCCCT	MIMAT0025967
Md-miR2118	CTACCGATGCCACTAAGTCCCA	MIMAT0026034
Md-miR7121f	TCCTCTTGGTGATCGCCCTGC	MIMAT0026045
Md-miR827	TTAGATGACCATCAACGAACA	MIMAT0026028
