# Supplementary material for: Md-miR156ab and Md-miR395 Target WRKY Transcription Factors to Influence Apple Resistance to Leaf Spot Disease
Source: Front Plant Sci. 2017 Apr 19;8:526. doi: 10.3389/fpls.2017.00526 (PMC5395612; doi:10.3389/fpls.2017.00526)
Supplement: Supplemental Table 2 — Sequences of the 19 differentially expressed novel mature miRNAs and their precursors. [file Table2.PDF]

Supplemental Table 2. Sequences of the 19 differentially expressed novel mature miRNAs and their precursors.

Novel miRNAs	miRNAs sequence	The precursor of miRNAs
mdm-miRN1	ATAAGTGGGTTTGTGGGCTGGCCC	ggcacatatgttggtattctattggaatgcaagtgggctggcttttgaacccatttatgggctattcaagtgggtaaaccataggttggtgcacttaaatggcctataagtgggtttgtgggctggccctcttgtatcctgatagtttagtatgatt
mdm-miRN2	AGGCAGTGGCTTGGTTAAGGG	taaaactcataggcagtggcttggttaagggaagccactggagccgtagtaaaagcaagtcttcaccgggcaattgtttatggacccgaa
mdm-miRN3	TGTGGGAATCTCTCTGATGCTT	tactaacaattagcaagtgatgatagaccagacagggacatctgtattctttttgaacatgatgatttgtctgtgggaatctctctgatgcttagatttcta
mdm-miRN4	GTTGGGAATCGAAGCATCACGA	tatttcctcgaagaagttaagtccagatgttagagctcaacaacttgcggcaatggatttatcaattgcccaagtccagtcttctcagcaagctgtttcgaaagattatcaagttatagggacctctttagcttccgaccgagtgcatttagaagagttaaagcgggagcgagagcagttgggaatcgaagcatcacgacttgaaggtgttcttttaaa
mdm-miRN5	CGATTCCCCAGCGGAGTCGCCA	gctgaatagatcgattccccagcggagtcgccaaaaatcgtgttggcaactttttgacaagtcgacaagcacgat
mdm-miRN6	CGGAATGAAGCTTACGAGAATG	atgaggcgggaattcatcgtaagcttcaattgaatgaacttgaggaaatacggaatgaagcttacgagaatgctcgacttt
mdm-miRN7	ACACCATCGCATCTCATGTTCC	ttctcgagaagagctgacatgcaactgagatgctaaggtgatggtattccaaaccaaccatgctatgtcatgttgaaaagttaaaacatatgacggtttgwgatgggcttgaaatgccaacaccatcgcatctcatgttccacgtaagttactctccact
mdm-miRN8	TCACCATTGCATCTCATGTTCC	ctctcgagaagagctaacttgtaactgagatgccaaggtgatagtatttcaaactaatcatgttattatgttgaaaagttgaaacataatggtgtctgacgggcttggaatgccatcaccattgcatctcatgttccatgtaagttaatctcatct
mdm-miRN9	CGAACTTATTGCAACTAGCTT	aagccaaagctctcaaatgcaagctagttgtaataagttcaatctttttatggccagtagtgccctaaataagcatcgaacttattgcaactagctttaatttttgagttttatgc
mdm-miRN10	CAGTCTGACAATATAACGTGC	gtataaaatgtatgtatatgtattaaaatgttcttttgccagccaatgtggcttgttatattgtcagattgtcaatctgaacctttaattaccattaatttacgtaattaatggttcagattgacagtctgacaatataacgtgccaaactgtttggcaattgagcattttcatacatatatatatatatat
mdm-miRN11	GCTCTCTATGCTTCTGTCATCA	ttgacagaagatagagagcactaatgatgatatgctaatttcattcagcaaaagcatctcacttcatttgtgctctctatgcttctgtcatca
mdm-miRN12	GTCGTTGTAGTATAGTGG	aggtaaaaatacttaccactatattacaacaacttgtttgtgaaattcaatggtaattttaaataataaatgttttaatattaccgatagcatttgattatatttttgttttaaagttacatttgattatatcgctatcggctataatgtaagtcatattcatccaatatcatgcaactttttgtatagatatattaatattactcgataatcatcaagtcgttgtagtatagtggtaagtattcccgcc
mdm-miRN13	ATTCAGTTGATGCAAGGCGGGATC	ctccgattcagttgatgcaaggcgggatccgattcaacgccggtaaggaaaccgtccatttcgcgccatccggcgcggtggtggaagcagaaccggttcccgacctgcaccaagcgaattagagacc
mdm-miRN14	AGCCGTAAACGATGGATACT	cgctgtgcgtatcgacccgtgcagtatccatcgtttaaagctaggggagcggaatgggattagataccccagtagtcctaagccgtaaacgatggatactgcacgggtcgatacggcacagcgctgtagctaacgcgtta
mdm-miRN15	GCTCTCTATGCTTCTGTCATC	atgttgtatgtatagtggagggtgatagtgtggttgctgacagaagatagagagcactaaggatgatatgcaaaacacacacatatatgtgcttctaattgtatttcatacttaacatcaatgttgatgtgaatcgtgaaaaaaaaaaaagagagaagagctagaggagcatctgcatctctattcctatgtgctctctatgcttctgtcatcaccttttttctctatttctctctacacattacatattcacat
mdm-miRN16	CGTTTCACGTCGGGTTCACC	cataattcaaataatccaaaaaaaataaaccaaataccttccacctcaagcgtttcacgtcgggttcaccaaatatgttgtattatgtgaagttttgattgagaatgcaccgagcacttggctatggaaggtaaaagaaaattgcaacaaaaagtcctaacaatccaataggattatagaaaatttataattaaacgttaacaatcataatcaaacaata
mdm-miRN17	TAAGTTCATCCAAACACCATA	gaataacaggtctcttctcaaagcaattgaagagtcaaggactcctatcactgcattttctgaaaataatgcatcttcaacgtggtgtttggatggaccttttaagatcctacaaaattttcaaagaaaagaaaattttggtactatttttacaaggtgaatcactcggaagtacaaaatcagaggaaatttttggtacttctcatggattgtaagatcttaataagttcatccaaacaccatattgaggatgcatagttttcagaaaatacaatgatacaagtctttgattcttctcttggaagctcattgtcatctatgaa
mdm-miRN18	TGCACACGGACTCGAGCTGCTCGGA	tgcacacggactcgagctgctcggagcacgtggtgtcgccgaggttcacgtgtgaggtgcaga
mdm-miRN19	ACAGGCGGGGGAACAAATATGAAT	acaggcgggggaacaaatatgaattttgctgagtcattagaattcatatttgttctaccgtcggtgg
