# Supplementary material for: Md-miR156ab and Md-miR395 Target WRKY Transcription Factors to Influence Apple Resistance to Leaf Spot Disease
Source: Front Plant Sci. 2017 Apr 19;8:526. doi: 10.3389/fpls.2017.00526 (PMC5395612; doi:10.3389/fpls.2017.00526)
Supplement: Supplemental Table 3 — Primer sequences used to clone the mature miRNAs. [file Tables3and4.PDF]

Supplemental Table 3. Primer sequences used to clone the mature miRNAs.

Name of miRNA	Reverse-transcription primers	Forward primer	Reverse primer
**Md-miR164**	gtcacatcgtatcgtgaagctgcgcagctgatgtgactgcacgtg	tgcactagcgtgtggagaagca	acatcgtatcgtgaag
**Md-miR396a**	gtcacatcgtatcgtgaagctgcgcagctgatgtgacctgttcaa	tgcactagcgtg ttccacagct	acatcgtatcgtgaag
**Md-miR396f**	gtcacatcgtatcgtgaagctgcgcagctgatgtgaccagttcaa	tgcactagcgtgttccacggct	acatcgtatcgtgaag
**Md-miR166**	gtcacatcgtatcgtgaagctgcgcagctgatgtgacggggaatg	tgcactagcgtgtcggaccagg	acatcgtatcgtgaag
**Md-miR156ab**	gtcacatcgtatcgtgaagctgcgcagctgatgtgacgatgacag	tgcactagcgtggctctctatg	acatcgtatcgtgaag
**Md-miR156x**	gtcacatcgtatcgtgaagctgcgcagctgatgtgacgtgctctc	tgcactagcgtgtgacagaaga	acatcgtatcgtgaag
**Md-miR395**	gtcacatcgtatcgtgaagctgcgcagctgatgtgacgagttccc	tgcactagcgtgctgaagtgtt	acatcgtatcgtgaag
**Md-miR159a**	gtcacatcgtatcgtgaagctgcgcagctgatgtgacggagctcc	tgcactagcgtgcttggattga	acatcgtatcgtgaag
**Md-miR159c**	gtcacatcgtatcgtgaagctgcgcagctgatgtgacgagctccc	tgcactagcgtgcttggattga	acatcgtatcgtgaag
**5srRNA**	gtcacatcgtatcgtgaagctgcgcagctgatgtgactggattgg	tgcactagcgtgtagaggaacc	acatcgtatcgtgaag

Supplemental Table 4. Primer sequences used to perform real time-PCR.

**Gene name**	Forward primer	Reverse primer
MdWRKYN1	CGCCAACTCTCCCTTCTCTTT	CACCCGAACTTTGCATATCTC
MdWRKY26	TCTCCATCCTCCTACTTTGCTATC	TTTACATTCTGTTGGTTGTTCTTCC
MdPR1	GGCTCAGTCCTTATCCAATCCTC	GCCTGCTACTTTGTCATCCCACG
MdPR2	CATTCGTCTAGATTATGCTCTTTCCACAG	TGTTGTTGCCGTCCCACCAGC
MdPR3-1	CTTTCGTTGCTGCTGCTCGGTC	TCTGGTGCACTTTCCCATCCTCC
MdPR3-2	CTAGTGGTACTTGCACGGGGCTG	GAAATCAACGCCGTCCAAAACC
MdPR4	CACCAGATGGTCCTTATGCATGGG	CCATAGTTGTAGTTGTGGGTGAGTTGG
MdPR5	GTAATGCTGCTGTGTGTAAACCC	AGAAAGCGACGACATAATCTGTG
MdGPX7	CTCGGTCTTTGGGTGTTTATG	GAAGAAGTCAAGCCACATCTTG
MdPR8	GCCACCGAAACTTACTCTCTCAC	CAACTCAATGTCGAAATCAACGC
MdAPX-1	CAAGGGTTGTGATCATTTGAGGG	CGAAGATAAGGGGGTTGGGAGTC
MdAPX-2	GGCGGGAGGAGTGGTGGTAGACG	CCAAACGAAGCATGATCGGAGCG
MdPR10-1	CACCTCCGTCATCCCCCCTGC	CCTTGTCAACCCCATCAATTCTGTG
MdPR10-2	GCAAACTACTCATACGCCTACAC	GCTCTTCCTTGATCTCAACATC
MdActin	TGACCGAATGAGCAAGGAAATTACT	TACTCAGCTTTGGCAATCCACATC
