# Supplementary material for: Md-miR156ab and Md-miR395 Target WRKY Transcription Factors to Influence Apple Resistance to Leaf Spot Disease
Source: Front Plant Sci. 2017 Apr 19;8:526. doi: 10.3389/fpls.2017.00526 (PMC5395612; doi:10.3389/fpls.2017.00526)
Supplement: Supplemental Table 5 — The disease rate of WT, EV, and OE-MdWRKYN1 and t-test analysis. Independent t-test demonstrated that there was significant difference (P < 0.01) in the disease rate of EV and OE-MdWRKYN1 under ALT1-inoculated. WT, non-infiltrated GD plants; EV, empty-vector (pFGC5941)-infiltrated plants; OE-MdWRKYN1, MdWRKYN1-overexpressing GD plants. [file Tables5-8.PDF]

Supplemental Table 5. The disease rate of WT, EV and OE-*MdWRKYN1* and *t*-test analysis.

| | WT | | | | EV | | | | OE-*MdWRKYN1* | | | | |
|---|---|---|---|---|---|---|---|---|---|---|---|---|---|
| Replicates | 1 | 2 | 3 | Mean % | 1 | 2 | 3 | Mean % | 1 | 2 | 3 | Mean% | *t*-test |
| Diseased leaves | 11 | 11 | 14 | | 11 | 12 | 13 | | 1 | 2 | 4 | | |
| Total leaves | 29 | 30 | 34 | 38.59 | 29 | 30 | 31 | 39.96 | 25 | 26 | 37 | 7.50 | 0.000142 (p<0.01) |
| Disease rate % | 37.93 | 36.67 | 41.18 | | 37.93 | 40 | 41.94 | | 4.00 | 7.69 | 10.81 | | |

Supplemental Table 6. The disease rate of WT, EV and STTM-miR156ab and *t*-test analysis.

| | WT | | | | EV | | | | STTM-miR156ab | | | | |
|---|---|---|---|---|---|---|---|---|---|---|---|---|---|
| Replicates | 1 | 2 | 3 | Mean % | 1 | 2 | 3 | Mean % | 1 | 2 | 3 | Mean % | *t*-test |
| Diseased leaves | 11 | 12 | 9 | | 13 | 12 | 13 | | 4 | 4 | 3 | | |
| Total leaves | 30 | 32 | 26 | 36.26 | 34 | 33 | 32 | 38.40 | 27 | 30 | 29 | 12.83 | 0.000143 (p<0.01) |
| Disease rate % | 36.67 | 37.50 | 34.61 | | 38.23 | 36.36 | 40.62 | | 14.81 | 13.33 | 10.34 | | |

Supplemental Table 7. The disease rate of WT, EV and OE-*MdWRKYN26* and *t*-test analysis.

| | WT | | | | EV | | | | OE-*MdWRKY26* | | | | |
|---|---|---|---|---|---|---|---|---|---|---|---|---|---|
| Replicates | 1 | 2 | 3 | Mean % | 1 | 2 | 3 | Mean % | 1 | 2 | 3 | Mean % | *t*-test |
| Diseased leaves | 10 | 12 | 10 | | 12 | 13 | 10 | | 2 | 2 | 1 | | |
| Total leaves | 29 | 32 | 32 | 34.41 | 34 | 34 | 31 | 35.26 | 39 | 33 | 28 | 4.92 | 0.000085 (p<0.01) |
| Disease rate % | 34.48 | 37.50 | 31.25 | | 35.29 | 38.23 | 32.25 | | 5.12 | 6.06 | 3.57 | | |

Supplemental Table 8. The disease rate of WT, EV and STTM-miR395 and *t*-test analysis.

| | WT | | | | EV | | | | STTM-miR395 | | | | |
|---|---|---|---|---|---|---|---|---|---|---|---|---|---|
| Replicates | 1 | 2 | 3 | Mean % | 1 | 2 | 3 | Mean % | 1 | 2 | 3 | Mean % | *t*-test |
| Diseased leaves | 10 | 12 | 10 | | 11 | 11 | 11 | | 4 | 5 | 5 | | |
| Total leaves | 29 | 34 | 30 | 34.37 | 30 | 29 | 31 | 36.69 | 28 | 29 | 32 | 15.71 | 0.000046 (p<0.01) |
| Disease rate % | 34.48 | 35.29 | 33.33 | | 36.67 | 37.93 | 35.48 | | 14.28 | 17.24 | 15.62 | | |
